# A simple, statistically robust test of discrimination

**DOI:** 10.1073/pnas.2416348122

**Published:** 2025-03-04

**Authors:** Johann D. Gaebler, Sharad Goel

**Affiliations:** ^a^Department of Statistics, Harvard University, Cambridge, MA 02138; ^b^Kennedy School of Government, Harvard University, Cambridge, MA 02138

**Keywords:** outcome tests, benchmark tests, inframarginality, monotone likelihood ratio property

## Abstract

In observational studies of discrimination, the most common statistical approaches consider either the rate at which decisions are made (benchmark tests) or the success rate of those decisions (outcome tests). Both tests, however, have well-known statistical limitations, sometimes suggesting discrimination even when there is none. Despite the fallibility of the benchmark and outcome tests individually, here we prove a surprisingly strong statistical guarantee: Under a common nonparametric assumption, at least one of the two tests must be correct; consequently, when both tests agree, they are guaranteed to yield correct conclusions. We present empirical evidence that the underlying assumption holds approximately in several important domains, including lending, education, and criminal justice—and that our hybrid test is robust to the moderate violations of the assumption that we observe in practice. Applying this approach to 2.8 million police stops across California, we find evidence of widespread racial discrimination.

## Significance

We introduce the robust outcome test, which suggests discrimination against a group if there are disparities in both outcome rates and decision rates. This hybrid test provides substantially stronger statistical guarantees than common alternatives while remaining simple to apply. On a dataset of California police stops, we find the standard outcome test indicates discrimination against White individuals in a third of the state’s large law enforcement agencies—defying expectations and calling into question the test’s findings of discrimination against racial minorities in many of the remaining agencies. In contrast, the robust outcome test uncovers consistent evidence of police discrimination against racial minorities across California. Our simple methodological improvement promises to strengthen the quality of evidence in statistical studies of discrimination.

When assessing claims of discrimination, researchers often begin by considering whether decision rates differ across groups defined by race or gender, typically after adjusting for relevant differences between groups. For example, to test for discrimination in banking, one might estimate differences in lending rates between White and Black loan applicants after adjusting for an individual’s credit score, income, and savings. Although such a “benchmark test” can be informative, it is prone to omitted-variable bias: Failing to adjust for all relevant information can yield misleading estimates. Nonetheless, benchmark tests have been applied in nearly every domain where discrimination is studied, generally under an implicit assumption that analysts have access to all relevant covariates (1–6).

To mitigate the omitted-variable problem inherent to benchmark tests, Becker (7, 8) introduced the “outcome test,” in which one looks not at decision rates but rather success rates. If, for example, loans issued to Black borrowers are repaid at higher rates than those issued to White borrowers, it suggests a double—and discriminatory—standard, with bank officials granting loans only to exceptionally creditworthy Black applicants. Owing perhaps to its simplicity and intuitive appeal, the outcome test has now become one of the most popular empirical approaches to detecting discrimination. Researchers have applied the test to audit a wide range of decisions, including lending, hiring, publication, and candidate election (9–15). The outcome test has gained particular prominence in criminal justice, among both researchers and policymakers (16–25).

**Fig. 1.**
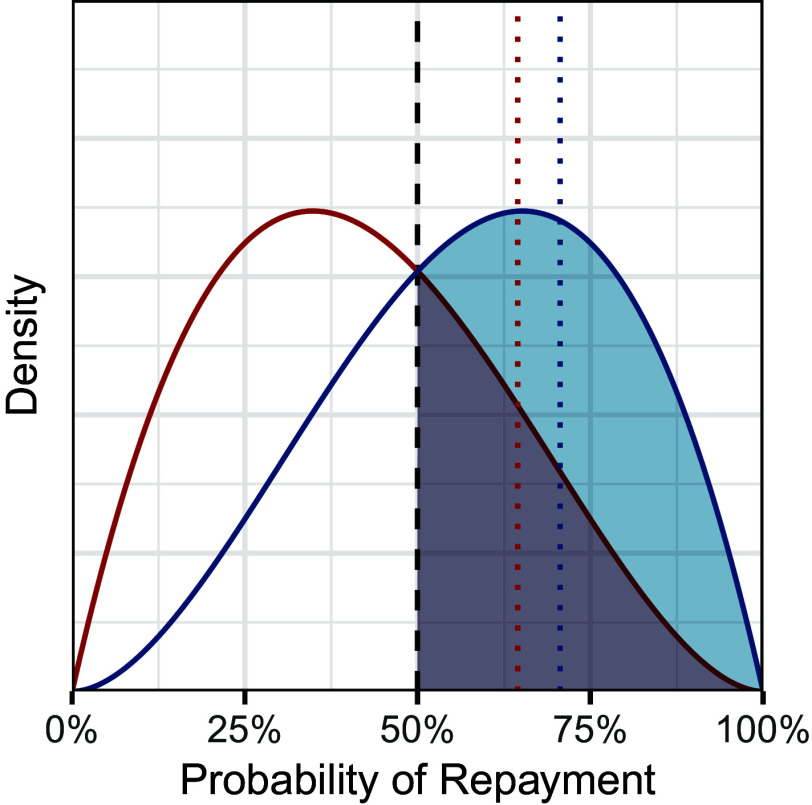
A stylized example illustrating the problem of inframarginality. The two curves depict the distribution of repayment probabilities for two hypothetical subpopulations. Applying a uniform lending threshold of 50% (dashed black vertical line) results in a higher repayment rate for loan recipients in the blue group (71%; dotted blue vertical line) than for recipients in the red group (64%; dotted red vertical line). The outcome test would thus incorrectly infer that members of the blue group were subjected to a more stringent lending standard.

Like the benchmark test, however, the outcome test suffers from well-known statistical limitations (26–31, 32). Consider the stylized example in Fig. 1, where the red and blue curves show the distribution of repayment probability across loan applicants in two different groups (henceforth, “risk distributions”). In this hypothetical, bank officials grant loans to those applicants who are at least 50% likely to repay their loans—indicated by the dashed black vertical line—irrespective of group membership. Despite this uniform lending standard, loan recipients in the blue group are more likely to repay their loans than recipients in the red group. In statistical terms, conditional on being above the lending threshold, the mean of the blue group’s risk distribution is greater than the mean of the red group’s. As a result, the outcome test would incorrectly conclude that applicants in the blue group were subject to a more stringent lending standard.

This problem of “inframarginality” has attracted considerable attention, prompting several attempts to place outcome tests on firmer statistical footing. Knowles et al. (32) developed a model of behavior under which risk distributions collapse to a single point, eliminating the possibility of inframarginality. Although theoretically interesting, the key assumption in that approach has been critiqued for being at odds with empirical evidence (33, 34). Anwar and Fang (31) proposed a test based on decision and outcome rates conditional on the race of both decision makers and those subject to those decisions. Their method is guaranteed, under certain conditions, to produce correct inferences, but it can only identify relative disparities between decision makers from different race groups. Building on that work, Alesina and La Ferrara (35) proposed a test of racial bias in capital sentencing based on the relative likelihood that decisions were overturned across defendant-victim race pairs. Arnold et al. (36) sidestepped concerns of inframarginality by directly estimating outcomes for individuals at the margin, leveraging quasi-random assignment of decision makers. Theirs is a statistically compelling approach but can only be applied in certain settings, where decision makers are plausibly quasi-randomly assigned and analysts have information on the actions of individual decision makers. Simoiu et al. (27) and Pierson et al. (37) worked to overcome inframarginality by simultaneously estimating risk distributions and decision thresholds with a parametric model. Their approach, however, is sensitive to the exact model form, and, in particular, estimates are not identified by the data alone. Finally, Jung et al. (34) used detailed individual-level information on covariates and outcomes to directly estimate group-specific risk distributions. The method is effective when it can be used (38–40), though the demanding data requirements limit the applicability of their approach.

Despite the limitations of both the benchmark and outcome tests, here we show that simply combining the two yields a robust outcome test with surprisingly strong statistical guarantees. In particular, if the group-specific risk distributions satisfy the monotone likelihood ratio property (MLRP) (41), then either the benchmark test—without adjusting for any covariates—or the standard outcome test must yield correct conclusions. Thus, when both the benchmark and outcome tests indicate discrimination, that conclusion must be correct. The MLRP is a widely applied assumption on signal distributions in information economics (e.g., refs. 42–45), as well as in the outcome test literature (e.g., refs. 31 and 46). We expect the MLRP to hold when it is similarly difficult to make accurate decisions for members of each group (e.g., when the group-specific risk distributions have similar variances, as in Fig. 1). Drawing on data from lending, education, and criminal justice, we present empirical evidence that the MLRP is approximately satisfied in several important domains. We further show that our hybrid test is robust to the moderate violations of the MLRP that we observe in our data. Applying this approach to 2.8 million police stops across 56 law enforcement agencies in California, we find evidence of pervasive discrimination in police searches of Black and Hispanic individuals—a pattern that would have been missed by the standard outcome test.

## 1. Statistical Guarantees

In our running lending example, our robust outcome test suggests discrimination against a group if two conditions hold simultaneously: 1) lending rates are lower for that group (the benchmark test), and 2) repayment rates among loan recipients are higher for the group (the standard outcome test). In the stylized example depicted in Fig. 1, loan recipients in the blue group have higher repayment rates, satisfying the standard outcome test; but members of the blue group are also more likely to receive loans, failing the benchmark test. In this case, whereas the standard outcome test incorrectly infers the blue group is held to a higher, discriminatory lending standard, our robust outcome test correctly concludes that there is insufficient evidence to support a claim of discrimination. We next present formal conditions under which the robust outcome test is guaranteed to produce correct results.

### 1.1. Formal Setup.

Our formal setup follows the literature on analyzing outcome tests (see, e.g., ref. 27). We imagine a population of individuals belonging to one of two groups G∈{0,1}, indicating, for example, their race or gender. Decision makers take a binary action D∈{0,1} for each individual, such as approving (D=1) or denying (D=0) an individual’s application for a loan. The decision maker is interested in some binary outcome Y∈{0,1}, which, in our running example, corresponds to loan repayment (e.g., Y=1 if the loan is repaid and Y=0 otherwise). The decision maker does not know Y at decision time, but they can estimate it based on the information X∈X—including group membership G—then available to them about the applicant. In particular, at the moment the decision is made, we assume they can estimate the probability that Y=1 given the available information:

[1] $$R\overset{\;\text{def}}{=}\mathrm{Pr}\left(Y=1|X\right).$$

In our running example, R is the decision maker’s estimate of the applicant’s repayment probability. Moreover, the conditional distributions of R by group correspond to the risk distributions in Fig. 1.

Finally, we assume that decision makers are rational, meaning that, within each group, their actions follow threshold rules. (This condition can be relaxed; see *SI Appendix*, section 1.B.) In particular, we assume they take action D=1 for individuals in group G=g if, and only if, R exceeds some (possibly group-specific) threshold $t_{g}$:

[2] $$\begin{array}{c} D\overset{\;\text{def}}{=}\left\{\begin{array}{cc} 1 & \;\text{if}\;G=g\;\;\text{and}\;t_{g}\leq R,\\ 0 & \;\text{otherwise}. \end{array}\right. \end{array}$$

Following Becker (7, 8), “discrimination” in this setting corresponds to having different group-specific thresholds (i.e., $t_{0}\neq t_{1}$), meaning decision makers apply a double standard. For instance, in our lending example, $t_{1}>t_{0}$ would mean that decision makers grant loans to members of group G=1 only if they are exceptionally qualified—amounting to discrimination against that group.^*^

With this setup, we now state our main technical result.

Theorem 1 *Suppose* Pr(G=1∣R=r) *is a monotonic function of* r, *and that, for* g∈{0,1}, *the conditional distribution of* R∣G=g *has positive density on* (0,1). *Now, if*:

1. 0<Pr(D=1∣G=1)<Pr(D=1∣G=0), *meaning that the decision rate is lower for group* G=1 *than group* G=0; *and*
2. Pr(Y=1∣D=1,G=1)>Pr(Y=1∣D=1,G=0), *meaning that the outcome rate is higher for group* G=1 *than group* G=0;

*then* $t_{0}<t_{1}$.

*Theorem* 1 shows that under the stated monotonicity assumption—which, we show in *SI Appendix*, is equivalent to the standard MLRP—a group with both lower decision rates and higher outcome rates is necessarily being held to a higher threshold. For ease of exposition, we present this result for risk distributions with positive densities, threshold decision rules, and binary outcomes, but a much more general version of the result holds. *SI Appendix*, Theorem S2 extends Theorem 1, removing the positive density assumption, allowing for quasi-rational decision makers (e.g., with decisions following a logistic curve rather than a threshold function), and incorporating real-valued outcomes (e.g., repayment amounts rather than a binary repayment indicator). See *SI Appendix*, section 1.A for the proof of *Theorem* 1 and *SI Appendix*, sections 1.B–1.E for the general case.

## 2. Assessing Monotonicity

The primary assumption of Theorem 1 is that Pr(G=1∣R=r) is monotonic—which, as discussed above, is equivalent to the group-specific risk distributions satisfying the MLRP. To build intuition about this nonparametric assumption, we consider related parametric conditions on the group-specific risk curves. In particular, a sufficient condition for monotonicity is that the group-specific risk curves are beta distributed with the same total count α+β (but possibly different means). More generally, monotonicity holds for betas if and only if the risk curves intersect exactly once, for instance as depicted in Fig. 1. (See *SI Appendix*, section 1.D for more general discussion of parametric conditions that ensure monotonicity and *SI Appendix*, Fig. S1 for an example where the MLRP fails.)

In our running example, equal total count roughly means that it is equally difficult for lenders to distinguish between high- and low-risk applicants across groups. One can imagine that an approximate version of this property holds not only in lending, but across many domains. Indeed, if it fails to hold, one might wonder whether decision makers are ignoring important features to mask discriminatory intent. With redlining, for example, lenders ignored key indicators of individual creditworthiness to justify denying loans to racial minorities (49).

### 2.1. Empirical Evaluation.

We explore the extent to which monotonicity holds in practice by considering group-specific empirical risk distributions in four domains, spanning banking, education, and criminal justice. Specifically, we consider: 1) the probability of default among applicants using an online financial technology platform, based on a bevy of traditional and nontraditional variables available to the platform when deciding whom to offer loans; 2) risk of recidivism among defendants awaiting court proceedings, as determined by COMPAS risk scores, which inform judicial bail decisions (50, 51); 3) the risk that pedestrians stopped by the police are carrying contraband, based on indicators such as the reason for the stop and the suspected offense, which inform officer decisions to search stopped individuals (1, 16); and 4) the probability that law school applicants will pass the bar exam, using their undergraduate grade-point average, LSAT score, and other information available to schools making admissions decisions (52). We observe only a proxy of the true outcome of interest—e.g., we see repayment outcomes only among those who received loans, not for the entire population of applicants. Similarly, we do not have the full suite of covariates available to decision makers. As a result, our estimates of risk are approximate. Nonetheless, these estimates give insight into the plausibility of the monotonicity assumption. (See *SI Appendix*, section 2 for details on the data sources and risk estimation methods.)

**Fig. 2.**
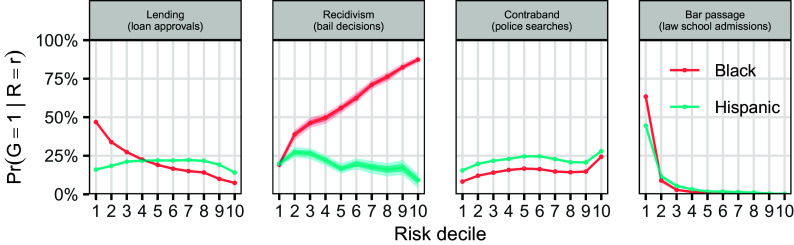
An empirical check of the monotonicity condition of Theorem 1 across four domains, comparing the risk distributions of Black vs. White (red) and Hispanic vs. White (blue) individuals. The estimated likelihood ratios all generally increase or decrease monotonically, providing evidence that the MLRP assumption often holds approximately in practice. The dark and light shaded bands (visible only in the recidivism panel) indicate 68% and 95% bootstrapped CIs, respectively.

For each of these four cases, we plot, in Fig. 2, Pr(G=1∣R=r) for Black vs. White individuals (red), and, separately, for Hispanic vs. White individuals (blue). (Here, “White” means non-Hispanic White.) We set G=1 for the smaller group in each comparison—which corresponds to Black or Hispanic individuals except for in our policing example, in which case White individuals are the smaller group. In every instance, we see that the monotonicity condition holds approximately, suggesting that it is, in practice, a relatively mild assumption. Monotonicity, however, does not hold exactly in these domains—nor would we expect it to in any real-world dataset. [Violations of the MLRP are visually apparent in Fig. 2, though formal tests of monotonicity could also be applied (53).] We thus next conduct a simulation study to assess the robustness of Theorem 1 to moderate violations of monotonicity, like those shown in Fig. 2.

### 2.2. Simulation Study.

Starting with the empirical risk distributions in the four examples considered above, we evaluate whether the robust and standard outcome tests correctly detect discrimination under a variety of discriminatory and nondiscriminatory scenarios. We find that across scenarios, the robust outcome test is nearly always correct: When it indicates discrimination against a group, that is almost always the correct inference. (Though, as expected, the test sometimes returns an inconclusive result.) In contrast, in these simulations, the standard outcome test often suggests discrimination against the group that in reality received preferential treatment.

For each of our four domains, we first generate synthetic datasets for Black and White individuals based on the estimated risk distributions. (See *SI Appendix*, Fig. S3 for Hispanic individuals.) To do so, for a given pair of hypothetical, group-specific decision thresholds $t_{g}$, we repeatedly draw individuals at random from each group, setting $D_{i}=1$ if their estimated risk $R_{i}$ exceeds $t_{g}$ and setting $Y_{i}=1$ with probability $R_{i}$. We then estimate the decision rate ${\hat{\;\text{DR}}}_{g}$ as the proportion of individuals in group G=g receiving positive decisions D=1; and we estimate the outcome rate ${\hat{\;\text{OR}}}_{g}$ as the proportion of individuals with positive outcomes Y=1, among those with positive decisions.^†^ Finally, we test for discrimination using the robust and standard outcome tests by comparing the decision and outcome rates across groups. In our simulations, we sweep $t_{g}$ across all percentiles of the risk distributions.^‡^

**Fig. 3.**
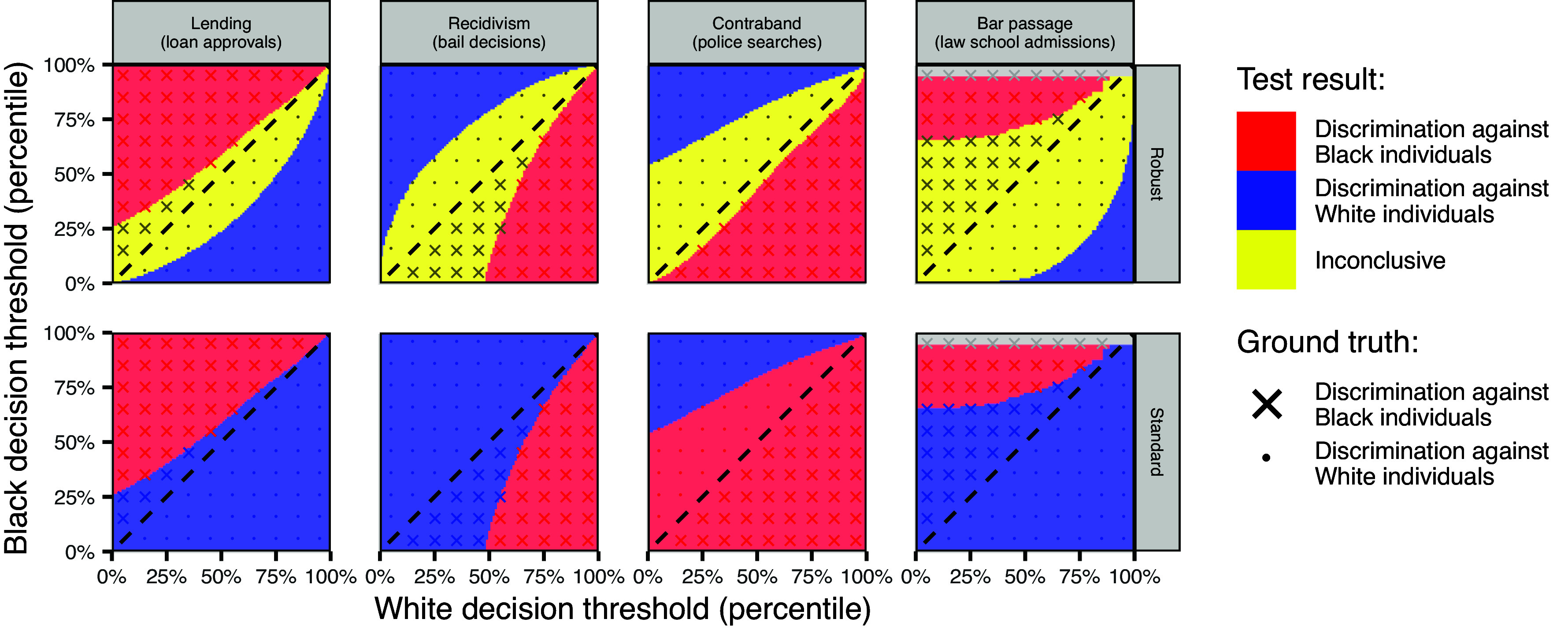
Results of a simulation study comparing the standard and robust outcome tests. The x-axis indicates the decision threshold for White individuals, and the y-axis indicates the decision threshold for Black individuals. The *Upper*-*Left* and *Lower*-*Right* triangular regions correspond to scenarios where decision makers discriminate against either Black or White individuals, as indicated by the “×” and “·” symbols, respectively; nondiscriminatory scenarios are shown by the dashed diagonal line. Red regions indicate where the tests suggest discrimination against Black individuals, blue regions indicate where the tests suggest discrimination against White individuals, and yellow regions indicate where the robust outcome test is inconclusive. The gray areas represent simulation scenarios that are not feasible because a threshold lies outside the support of the risk distribution of the corresponding group. Across simulations, the standard outcome test indicates discrimination when, in reality, there is none—and often indicates discrimination against the group that in actuality was favored. In contrast, the robust outcome test is nearly always directionally accurate, though it sometimes returns an inconclusive result.

The results of the simulation are shown in Fig. 3. As can be seen in the *Top* panels, the robust outcome test is virtually always inconclusive in the absence of discrimination—as we would hope—shown by the yellow region covering the diagonal “no discrimination” line. Moreover, in the off-diagonal regions, where the group-specific thresholds differ, the robust outcome test frequently detects discrimination, and nearly always in the right direction. In contrast, the standard outcome test, as shown in the *Bottom* panels of Fig. 3, makes frequent errors, both suggesting discrimination when there is none, as well as indicating discrimination against the group that, in actuality, decision makers favored. Thus, even in these cases where the MLRP does not hold exactly, the robust outcome test still provides correct inferences, and, moreover, outperforms the standard outcome test.

The extent to which the robust outcome test is able to detect discrimination—as opposed to returning an inconclusive result—varies across domains. In particular, when there is a large gap in base rates, it is hard to detect instances of discrimination. In these cases, even when there is (modest) discrimination, the higher base rate group still tends to have both the higher decision rate and the higher outcome rate, yielding an inconclusive result under the robust outcome test. Accordingly, when there is a large gap between base rates, the robust outcome test can only definitively detect more severe instances of discrimination. For instance, the law school admissions example in Fig. 3 features especially large gaps between the base rates of different groups, resulting in a large inconclusive region. See *SI Appendix*, section 3.3 and *SI Appendix*, Fig. S7 for further discussion of the impact of differences in base rates on the sensitivity of the robust outcome test.

In our formal analysis and simulations above, we assume that decision makers are rational within groups, making decisions based on a (potentially group-specific) threshold. In *SI Appendix*, section 1.B, we relax this assumption and consider quasi-rational decision makers. Simulation results for quasi-rational decision makers show similar patterns; see *SI Appendix*, Figs. S5 and S6.

## 3. An Application to Police Stops

We conclude our analysis by applying the robust outcome test to data on 2.8 million police stops conducted in 2022 by 56 law enforcement agencies across California. These data were collected as part of California’s Racial Identity and Profiling Act (RIPA) (38, 54). After an individual is stopped by the police, officers may legally conduct a search of the individual or their vehicle if there is sufficient evidence that the individual possesses contraband. Here we use the robust outcome test to determine whether officers apply the same standard of evidence across racial groups when deciding whom to search.^§^ To do so, we compute, for each jurisdiction, the race-specific search rates and search success rates (i.e., the proportion of searches that resulted in recovery of contraband). If members of a group are both searched more often and those searches turn up contraband less often, then the robust outcome test indicates that the group was searched according to a lower, discriminatory standard of evidence. (In contrast to our running lending example, where we equated discrimination with a higher lending threshold, discrimination here corresponds to a lower search threshold.)

In the RIPA data, individual-level covariates are recorded selectively (e.g., many covariates are only recorded when a search is conducted). This missingness makes estimating risk difficult (38), and, consequently, we cannot directly validate the MLRP assumption. We expect, however, that the robust outcome test is most useful in precisely these data-limited settings. [If one could accurately estimate risk, other methods may be more appropriate, e.g., risk-adjusted regression (34).] Absent direct evidence of the MLRP, the empirical results of Section 2—and, in particular, the monotonicity observed in the closely related policing domain considered there—offer reasonable assurances that the MLRP holds approximately for the RIPA data.

**Fig. 4.**
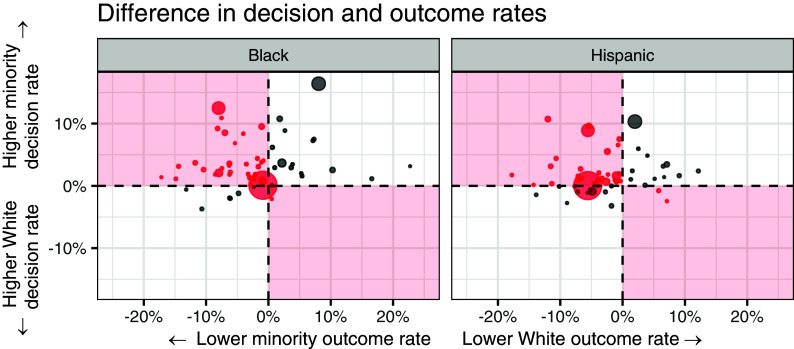
An illustration of the robust outcome test applied to 56 law enforcement agencies across California, with points corresponding to agencies and sized by the number of stops. In each panel, the robust outcome test suggests agencies in the *Upper*-*Left* quadrant discriminated against racial minorities when deciding whom to search, and that agencies in the *Lower*-*Right* quadrant discriminated against White individuals. The test yields inconclusive results for agencies in the white quadrants on the diagonal.

We plot the results of our empirical analysis in Fig. 4, with points corresponding to agencies, sized by the number of recorded stops. Each panel compares stops of White individuals to those of racial minorities (Black or Hispanic individuals, respectively). In each panel, differences between group-specific search rates are plotted on the vertical axis, and differences in search success rates on the horizontal axis. Under the robust outcome test, the red quadrants thus indicate racial discrimination, as those regions contain jurisdictions with both higher search rates and lower search success rates for one of the groups. In particular, the upper-left quadrants suggest discrimination against racial minorities, and the lower-right quadrants suggest discrimination against White individuals. The robust outcome test returns an inconclusive result for agencies in the white, diagonal quadrants, as those correspond to both higher search rates and higher search success rates for one of the groups.

Of the 56 agencies we consider, the robust outcome test suggests discrimination against Black individuals by 33, and discrimination against Hispanic individuals by 32. The test returns an inconclusive result in nearly all of the remaining cases.^¶^ The robust outcome test thus suggests a pattern of widespread discrimination against racial minorities in police searches across California.

The standard outcome test, in contrast, suggests White individuals were searched according to a lower standard of evidence than Black individuals in about one-third of agencies—corresponding to points in the right-hand quadrants—indicating discrimination against White individuals in those jurisdictions. While not impossible, that result is at odds with an extensive analysis of police discrimination in the literature (1, 16, 17, 22, 24, 27, 34, 38, 55–57), pointing to the statistical limitations of the standard outcome test. Due to this lack of face validity, it is easy to dismiss results from the standard outcome test even when it suggests more plausible findings of discrimination against racial minorities, illustrating the value of our robust alternative.

## 4. Discussion

Our empirical analysis of police decisions suggests that the robust outcome test is, in practice, a more accurate barometer of bias than the standard outcome test. Further, it is a logistically straightforward and intuitively appealing method for assessing discrimination. Applying the test requires knowing only group-specific decision and success rates, information that is often readily available in administrative databases. Critically, the robust outcome test avoids omitted-variable bias because it does not use individual-level covariates. Nor does it require decision maker demographics, as other methods do (e.g., refs. 31 and 34) but which—like detailed covariate information—administrative records often omit.^#^ Further—and in contrast to both the benchmark and standard outcome tests—the robust outcome test is guaranteed to produce correct results under a realistic assumption about the underlying risk distributions. Compared to more statistically sophisticated approaches like the threshold test (27, 37), it requires substantially weaker assumptions to establish correctness.

Our theoretical and empirical results strengthen several past findings in the outcome test literature, where decision rates were reported and are consistent with outcome rates (e.g., refs. 17, 19, 20, and 22–25). (In these instances, decision rates were reported incidentally or analyzed separately from outcome rates, rather than in the hybrid fashion we suggest.) In many cases, however, researchers simply apply the standard outcome test without reporting decision rates (e.g., refs. 9, 10, 12–15, and 21). Our results thus highlight an important gap in the literature, and suggest a straightforward change to improve current methodological practice.

Despite the benefits of our robust outcome test, it is important to recognize its limitations. First, and most importantly, our proof of correctness rests on a key monotonicity assumption. We presented empirical evidence that this assumption holds approximately in many common cases, and we further showed that, in practice, we obtain correct inferences even when monotonicity does not hold exactly. But the test may yield incorrect results in settings where it is substantially easier to make inferences about one group than another (*SI Appendix*, Fig. S1). Second, like the standard outcome test, computing success rates requires unbiased outcomes. In the policing data we analyzed, it seems likely that our main outcome of interest—contraband recovery—was generally recorded accurately, but that may not always be the case. Third, our robust outcome test can return inconclusive results. In these cases, an absence of evidence of discrimination may stem either from a lack of actual discrimination or from real discrimination that has gone undetected. In particular, as discussed above, the robust outcome test often fails to detect small or moderate threshold gaps when there is a large difference in base rates between groups. We note, though, that in our empirical analysis of police stops, the robust outcome test produced conclusive results in the majority of instances, revealing a pervasive pattern of discrimination. Finally, the robust outcome test—like the standard outcome test—formally produces only a binary determination of discrimination, not a continuous measure of the degree of discrimination.

These limitations suggest promising avenues for future work. In practice, we suspect that greater gaps in decision and success rates point toward greater discrimination. However, rigorously grounding this intuition requires both choosing an appropriate continuous measure of discrimination and, likely, additional parametric assumptions. Relatedly, with stronger, parametric assumptions, one can likely develop variants of the robust outcome test that more often return conclusive results. Lastly, the robust outcome test requires only aggregate information on decision and outcome rates—one of its strengths—but estimates can likely be improved by appropriately leveraging individual-level covariates when they are available. We caution, though, that simply conditioning on the available information can lead to “included-variable bias” (34, 48), masking discrimination.^‖^

Recent years have brought renewed urgency to identifying and ameliorating bias in policing and beyond. We hope our work helps further this area of study, both by providing a straightforward and statistically robust method for detecting discrimination, and by offering a blueprint for formally studying empirical tests of bias.

## Supplementary Material

Appendix 01 (PDF)

## Data Availability

Data and analysis code are available on GitHub (58).

## Footnotes

^*^As is typical of statistical tests of discrimination, our definition cannot differentiate intentional and unintentional behavior. For instance, different lending thresholds could result from animus, or what is commonly called “disparate treatment” (47). Alternatively, threshold gaps might arise unintentionally, potentially leading to “disparate impact”—unjustified, avoidable disparities (38). For example, suppose a bank applies a uniform lending threshold to miscalibrated estimates of risk, which consistently underestimate repayment probability for members of one group. Such miscalibration could occur if the bank does not account for varying relationships between covariates and repayment across groups—sometimes called the “subgroup invalidity” problem (48). Our definition of discrimination considers the bank’s behavior relative to the true (necessarily calibrated) risk in Eq. **1**. In terms of this risk, the bank’s behavior amounts to applying a double standard, unintentionally but favorably lending to the group whose risk it underestimated. Despite this inherent uncertainty about the underlying mechanism, statistical results like those produced by the robust outcome test can bolster legal claims of both disparate treatment and disparate impact (24).

^†^As the size of the synthetic datasets goes to infinity, the limits of ${\hat{\;\text{DR}}}_{g}$ and ${\hat{\;\text{OR}}}_{g}$ can be computed directly from the risk distributions, without sampling. We take this analytic approach in our simulations for computational efficiency.

^‡^Specifically, we use the quantiles of the average risk distribution across groups, i.e., the distribution with CDF $F(x)\overset{def}{=}\frac{1}{3}[F_{\text{B}}(x)+F_{\text{H}}(x)+F_{\text{W}}(x)]$, where $F_{g}\left(x\right)$ denotes the CDF of Black, Hispanic, and White individuals, respectively. This parameterization ensures that thresholds are not sensitive to the relative sizes of the groups. We exclude the 0th and 100th percentiles.

^§^Following the outcome test literature (e.g., ref. 17), here we consider only potential discrimination in search decisions—and not in, for example, stop decisions. The main advantages of focusing on search decisions in our setting are two-fold. First, it is clearer what constitutes “success” (i.e., recovery of contraband), facilitating the computation of outcome rates. Second, the pool of individuals subject to the search decision (i.e., individuals stopped by officers) is well defined, facilitating computation of decision rates. In contrast, there is more ambiguity in specifying the set of individuals subject to stop decisions. One could, for example, consider the residential population or, alternatively, the daytime population, and could additionally choose to weight either of these populations by their time outdoors. Compounding this definitional challenge, it can be hard to reliably quantify the size and demographic composition of several of these variants.

^¶^In these inconclusive instances, the standard outcome test generally suggests discrimination against White individuals. In contrast, in the policing simulation shown in Fig. 3, when the robust outcome test is inconclusive, the standard outcome test generally suggests discrimination against Black individuals. This difference may stem in part from the risk distributions being broadly similar in both our RIPA analysis and policing simulation, but with the high and low base rate groups reversed. In particular, in the policing dataset used for the simulation, stopped White pedestrians are the higher base rate group—likely because of discrimination in the initial stop decisions (16). In the RIPA data—primarily consisting of motor vehicle stops, which may be less prone to discrimination than pedestrian stops (17)—stopped White individuals are likely the lower base rate group (38).

^#^The RIPA data, in addition to the covariate shortcomings noted above, do not have officer demographics, complicating statistical analyses of discrimination that seek to leverage such information.

^‖^For example, imagine that officers apply a lower bar for searching pedestrians in neighborhoods with a large number of racial minorities—but apply uniform search standards within neighborhoods, regardless of an individual’s race. Then, by conditioning on neighborhood, we might correctly infer there is no discrimination within neighborhoods while failing to capture the larger pattern.
