# Supplementary material for: A simple, statistically robust test of discrimination
Source: Proc Natl Acad Sci U S A. 2025 Mar 4;122(10):e2416348122. doi: 10.1073/pnas.2416348122 (PMC11912395; doi:10.1073/pnas.2416348122)
Supplement: Supplementary file 1 — Appendix 01 (PDF) [file pnas.2416348122.sapp.pdf]

Supporting Information for

A Simple, Statistically Robust Test of Discrimination

Johann D. Gaebler and Sharad Goel

Johann D. Gaebler.
E-mail: jgaebler@fas.harvard.edu

This PDF file includes:

Supporting text
Figs. S1 to S7
SI References

Supporting Information Text

1. Mathematical Appendix

We structure the mathematical theory of the robust outcome test in six parts. First, in SI A, to provide intuition for the general result, we prove Theorem 1. Then, in SI B, we extend the formal problem in the main text to a more general setting that accommodates arbitrary utilities and quasi-rational decision makers before stating Theorem S2, a generalization of Theorem 1. In SI C, we give an overview of important properties of risk-decision curves, a key theoretical object introduced in our general setting; in particular, we show that risk-decision curves are well-defined and that the notion of generation, introduced in SI B, is also well-defined and non-vacuous. Next, we revisit important properties of stochastic orderings in SI D, including stochastic dominance and the MLRP. We prove Theorem S2 in SI E. Finally, in SI F, we give an overview of how to account for sampling error in the robust outcome test.

A. Proof of Theorem 1. We begin by noting that Theorem 1 cannot hold without *some* hypothesis on the risk distributions. Figure S1 illustrates one possible failure of the robust outcome test in our running lending example. Here, there is no discrimination, because lending decisions are made according to a uniform threshold. However, lending rates are lower and repayment rates higher for the blue group. One intuitive way of capturing the issue is that it is easier for loan officers to determine whether an applicant will default in the blue group than in the red group because the risk distribution of the blue group has higher variance than that of the red group. The MLRP formalizes and generalizes this intuition, capturing the key properties needed for the robust outcome test to be correct.

First, we note that the MLRP is equivalent to the—in our setting, more intuitive—monotone conditional probability (MCP) condition stated in Theorem 1. Suppose that the conditional distributions of $R \mid G = g$ have positive densities on $(0, 1)$ given by $f_{R|G=g}(r)$. The MLRP simply states that the likelihood ratio

$$\frac{f_{R|G=1}(r)}{f_{R|G=0}(r)}$$

is a monotonic function of r. Recalling the monotonicity condition in Theorem 1, observe that

$$g(r) \overset{\text{def}}{=} \Pr(G = 1 \mid R = r) = \frac{p \cdot f_{R|G=1}(r)}{(1-p) \cdot f_{R|G=0}(r) + p \cdot f_{R|G=1}(r)},$$

where $p \overset{\text{def}}{=} \Pr(G = 1)$. Note that if

$$h(q) \overset{\text{def}}{=} \frac{1-p}{p} \cdot \frac{q}{1-q},$$

then

$$(h \circ g)(r) = \frac{f_{R|G=1}(r)}{f_{R|G=0}(r)},$$

i.e., the likelihood ratio. As a consequence, since $h(q)$ is monotonically increasing on $(0, 1)$, the MLRP holds in this case if and only if $\Pr(G = 1 \mid R = r)$ is a monotonic function of r.

To understand how the MLRP connects to the proof of Theorem 1, let $g^{\text{lwr}} \in \{0, 1\}$ denote the lower "base rate" group and $g^{\text{upr}} \in \{0, 1\}$ the higher base rate group, i.e., g^{lwr} and g^{upr} are such that

$$\Pr(Y = 1 \mid G = g^{\text{lwr}}) \leq \Pr(Y = 1 \mid G = g^{\text{upr}}).$$

(For avoidance of doubt, "base rate" refers throughout to the unconditional outcome rate of a group, not the prevalence of a group.) Satisfying the MLRP implies the following two useful properties. First, the risk-distribution of group $G = g^{\text{upr}}$ is "right-shifted" relative to that of group $G = g^{\text{lwr}}$, i.e.,

$$\Pr(R \geq r \mid G = g^{\text{upr}}) \geq \Pr(R \geq r \mid G = g^{\text{lwr}}) \quad \text{for all } r \in [0, 1]; \tag{3}$$

that is, the MLRP implies that the distributions also satisfy stochastic dominance. Secondly, the lower base rate group retains a lower outcome rate even after conditioning on risk being above some threshold $t < 1$. More specifically,*

$$\Pr(Y = 1 \mid G = g^{\text{lwr}}, t \leq R) \leq \Pr(Y = 1 \mid G = g^{\text{upr}}, t \leq R). \tag{4}$$

See Theorems 1.C.1 and 1.C.5 in Shaked and Shantikumar (1) for proof of these properties.

This is enough to allow us to prove Theorem 1.

* In general, the MLRP implies—and is equivalent to—uniform conditional stochastic dominance; here we derive Eq. (4) from uniform conditional stochastic dominance using the fact that, by the law of iterated expectations and the definition of R,

$$\Pr(Y = 1 \mid G = g, t \leq R) = \mathbb{E}[\Pr(Y = 1 \mid X) \mid G = g, t \leq R] = \mathbb{E}[R \mid G = g, t \leq R].$$

Proof of Theorem 1. We proceed by proving the contrapositive: if $t_1 \leq t_0$, then either the decision rate of group $G = 0$ will be no larger than that of group $G = 1$, or the outcome rate will be no smaller, i.e.,

$$\Pr(D = 1 \mid G = 0) \leq \Pr(D = 1 \mid G = 1) \qquad [5]$$

or

$$\Pr(Y = 1 \mid G = 0, D = 1) \geq \Pr(Y = 1 \mid G = 1, D = 1). \qquad [6]$$

We will show that, depending on whether $G = 0$ is the lower base rate group or the higher base rate group, either the benchmark test in Eq. (5) or the outcome test in Eq. (6), respectively, will point in the correct direction.

Suppose that group $G = 0$ has the *lower* base rate. Eq. (3) implies that

$$\Pr(R \geq t_0 \mid G = 1) \geq \Pr(R \geq t_0 \mid G = 0).$$

Furthermore, reducing the decision threshold from t_0 to t_1 can only increase the decision rate for group $G = 1$. Thus, in this case, the decision rate for group $G = 0$ cannot exceed that of group $G = 1$, i.e.,

$$\Pr(D = 1 \mid G = 1) = \Pr(R \geq t_1 \mid G = 1) \geq \Pr(R \geq t_0 \mid G = 0) = \Pr(D = 0 \mid G = 0),$$

showing that Eq. (5) holds.

On the other hand, suppose that group $G = 0$ has the *higher* base rate. The outcome test looks at the outcome rates of the groups *after* conditioning on receiving a positive decision. More specifically,

$$\Pr(Y = 1 \mid G = g, D = 1) = \Pr(Y = 1 \mid G = g, t_g \leq R)$$

by the definition of D in Eq. (2). Now, since $\Pr(D = 1 \mid G = g) > 0$, it follows that $t_g < 1$ for $g \in \{0, 1\}$. Therefore, by Eq. (4), we have that

$$\Pr(Y = 1 \mid G = 0, t_0 \leq R) \geq \Pr(Y = 1 \mid G = 1, t_0 \leq R).$$

Again, lowering the decision threshold from t_0 to t_1 only reduces the outcome rate for group $G = 1$, i.e.,

$$\Pr(Y = 1 \mid G = 0, D = 1) \geq \Pr(Y = 1 \mid G = 1, D = 1),$$

showing that Eq. (6) holds, and completing the proof. □

In proving Theorem 1, the key insight is that, under the MLRP, whether group $G = 0$ is the lower or higher base rate group, either the benchmark or the standard outcome test will correctly detect the relative ordering of t_0 and t_1—though we do not know which one. As a result, when both tests indicate discrimination against the same group, the conclusion is unambiguous. Theorem S2 below extends this argument to a much more general setting. The chief technical obstacles there are: (1) showing that the standard outcome test still points in the right direction for the higher base rate group, and (2) accounting for quasi-rational decision makers and more complex risk distributions without densities.

B. General Utilities and Quasi-Rational Decision Makers. The correctness of the robust outcome test holds in a more general setting than the one presented in Section 1 that allows for both quasi-rational decision makers, as well as more general bases for their decisions. The general theorem and its assumptions are most naturally presented in the language of measure theory, which we adopt throughout.

We again imagine a population of individuals belonging to one of two groups $G \in \{0, 1\}$. To avoid trivialities, we assume that neither group is empty, i.e.,

$$\Pr(G = g) > 0 \qquad \text{for} \quad g \in \{0, 1\}. \qquad [7]$$

As above, we assume that decision makers make binary decisions $D \in \{0, 1\}$ for each individual. We also assume that a non-zero proportion of individuals in each group receives decision $D = 1$, i.e.,

$$\Pr(D = 1 \mid G = g) > 0 \qquad \text{for} \quad g \in \{0, 1\}. \qquad [8]$$

In our running lending example, loan officers base decisions on an applicant's probability of repayment, i.e., on $U \stackrel{\text{def}}{=} \Pr(Y = 1 \mid X)$, where $Y = 1$ denotes repayment of the loan and X denotes loan applicants' observable features when lending decisions occur. In a more realistic model, instead of directly on $\Pr(Y = 1 \mid X)$, loan officers might instead base their decisions on a calibrated risk score U. We can further generalize to an arbitrary utility U, dissociating from a particular outcome Y and covariates X. For example, U could represent the lender's expected return on lending to an applicant, rather

than just the applicant's probability of default. However, for consistency with Section 1, we still refer to distributions of U as *risk* distributions. We assume that the expectation of U is well-defined, i.e.,

$$\mathbb{E}[|U|] < \infty. \quad [9]$$

To ensure that decisions can be compared across groups, we assume the following analogue of the common overlap assumption (2):

$$0 < \Pr(G = g \mid U) < 1 \quad \text{a.s.} \quad [10]$$

In contrast to the usual setting in the outcome test literature, we do not require there to be a single decision maker—or, more generally, a collection of identical decision makers—making decisions based on group-specific thresholds t_g, $g \in \{0, 1\}$. Instead, we capture the decision process through *risk-decision curves*:

$$d_g(u) \stackrel{\text{def}}{=} \Pr(D = 1 \mid U = u, G = g). \quad [11]$$

The risk-decision curves encode the proportion of individuals who receive a positive decision among those who belong to group $G = g$ with utility $U = u$. We can ask whether one group receives positive decisions more frequently at every level of utility, representing a double-standard. Depending on whether a positive decision is "desirable," as in lending, or "undesirable," as in policing, we understand $d_g(u) < d_{g'}(u)$ as either discrimination or preferential treatment.

In Section 1, we assumed particularly simple risk-decision curves of the following form:

$$d_g(u) = \mathbf{1}(u \geq t_g),$$

where $\mathbf{1}(\cdots)$ denotes an indicator function. Here we generalize beyond threshold rules to cases in which the decision makers collectively exhibit a form of bounded rationality.

Definition S1 (Bounded Rationality)**.** We say that the risk-decision curve $d_g(u)$ exhibits *bounded rationality* when it is right-continuous and non-decreasing U-a.s.[†]

The definition of bounded rationality itself is very general, although the proof of our main theorem requires an additional restriction defined below on the risk-decision curves. In practice, we expect risk-decision curves to be continuous; however, requiring only right continuity allows for the possibility that there are thresholds where decision makers have a discontinuous increase in their probability of choosing $D = 1$, as, e.g., would be the case for rational decision makers at the threshold t_g.

Since it is a.s. bounded between zero and one, a risk-decision curve $d_g(u)$ exhibiting bounded rationality can be seen as the cumulative distribution function (CDF) of a distribution H_g on the extended real numbers $\bar{\mathbb{R}} = \mathbb{R} \cup \{-\infty, \infty\}$ where

$$\Pr(H_g = -\infty) = \lim_{u \to -\infty} d_g(u), \quad \Pr(H_g \leq t) = d_g(t), \quad \text{and} \quad \Pr(H_g = \infty) = \lim_{u \to \infty} 1 - d_g(u). \quad [12]$$

We say that $d_g(u)$ *generates* H_g. Because $d_g(u)$ is defined only U-a.s., $d_g(u)$ may not generate a *unique* distribution H_g. But, for our purposes, the different generated distributions are largely interchangeable, and so we will often refer to *the* generated distribution H_g. (For the well-definedness of H_g and related considerations, see SI C below.)

The final condition we require is that the risk-decision curves generate some pair of distributions satisfying the MLRP. This property holds if, e.g., the risk-decision curves are threshold rules. (See SI D below for discussion of the MLRP and non-continuous distributions.) We now state the general version of our main result.

Theorem S2. *Suppose that the following two conditions hold:*

- $\Pr(G = 1 \mid U = u)$ *is U-a.s. monotone,*
- *The risk-decision curves $d_g(u)$ for $g \in \{0, 1\}$ generate distributions satisfying the MLRP.*

Under these conditions, if

$$\Pr(D = 1 \mid G = 0) > \Pr(D = 1 \mid G = 1),$$

and

$$\mathbb{E}[U \mid D = 1, G = 0] < \mathbb{E}[U \mid D = 1, G = 1],$$

then $d_0(u) \geq d_1(u)$ U-a.s., where the equality is strict with positive probability.

[†] By "U-a.s.," we mean that a property holds for all $u \in \mathbb{R} \setminus S$ where $\Pr(U \in S) = 0$.

We defer the proof to SI E. As with threshold decision rules, the robust outcome test holds in this more general setting even under the modest violations of the MLRP that we see in practice; see SI 3 for the results of a simulation study analogous to the one presented in Section 2.B above.

Theorem S2 assumes that the risk-decision curves generate distributions satisfying the MLRP. This property holds in a variety of settings, including when decisions are made according to risk thresholds t_g as in Section 1. It is also satisfied by logistic risk-decision curves of the following form:

$$d_g(u) = \frac{1}{1 + \exp(\lambda \cdot [t_g - u])}. \qquad [13]$$

Here, as in the bounded rationality literature (e.g., ref. 3), $\lambda > 0$ represents the decision makers' degree of "rationality," with $\lambda \to \infty$ recovering threshold decision rules in the limit; and t_g represents a "soft" threshold at which decision makers become more likely to make decision $D = 1$ than $D = 0$. Many other families of risk-decision curves satisfy the MLRP, such as the CDFs of normal distributions with the same variance or beta distributions with the same total count. More generally, the CDFs of log, logit, or other monotonic transformations of normal, beta, or gamma distributions whose densities cross once satisfy the MLRP. (See SI D below for more detailed discussion of families of distributions satisfying the MLRP, and Figure S4 for an illustration of logit-normal CDF risk-decision curves satisfying the MLRP.) Intuitively, risk-decision curves satisfy our assumption when, as in Eq. (13), decision makers are similarly sensitive to risk across groups—a property that is strictly weaker than the "rationality" generally assumed in the outcome test literature.

C. Risk-Decision Curves. We begin by noting that risk-decision curves are well-defined. Recall the definition in Eq. (11):

$$d_g(u) \stackrel{\text{def}}{=} \Pr(D = 1 \mid U = u, G = g).$$

The risk-decision curves $d_g(u)$ exist and are well-defined U-a.s. by the Doob-Dynkin lemma because of the non-triviality assumptions in Eqs. (7) and (8) and the overlap assumption in Eq. (10).

The following lemma shows that the definition of generation is not vacuous.

Lemma S3. *Any risk-decision curve $d_g(u)$ exhibiting bounded rationality generates some distribution H_g.*

Proof. First, we recall that $d_g(u)$ is non-decreasing, bounded between zero and one, and right-continuous U-a.s. In other words, there exists a U-null set S such that these properties hold for all $u \in \mathcal{R} \stackrel{\text{def}}{=} \mathbb{R} \setminus S$. For $k \in \{1, \ldots, 2^n\}$, let

$$\ell(k, n) \stackrel{\text{def}}{=} \inf \left\{ u \in \mathcal{R} : d_g(u) \geq k \cdot 2^{-n} \right\}, \qquad [14]$$

and define $F_n : \mathbb{R} \to [0, 1]$ as follows:

$$F_n(u) \stackrel{\text{def}}{=} \frac{1}{2^n} \sum_{k=1}^{2^n} \mathbf{1}(\ell(k, n) \leq u). \qquad [15]$$

The function $F_n(u)$ approximates $d_g(u)$. In particular, for all $u \in \mathcal{R}$,

$$0 \leq d_g(u) - F_n(u) < 2^{-n}. \qquad [16]$$

To see why, suppose that $u^* \in \mathcal{R}$ is arbitrary and satisfies

$$(k - 1) \cdot 2^{-n} \leq d_g(u^*) < k \cdot 2^{-n}.$$

Then it immediately follows from Eq. (14) that $\ell(k - 1, n) \leq u^*$. (If $k = 1$, then $\ell(k - 1, n)$ is, strictly speaking, not defined by Eq. (14), but the proof differs only in one minor detail noted below.) Separately, by right continuity, there exists $\delta > 0$ such that for all $u \in [u^*, u^* + \delta) \cap \mathcal{R}$,

$$d_g(u) < k \cdot 2^{-n}.$$

By monotonicity, for all $u \in \mathcal{R}$ less than or equal to u^*,

$$d_g(u) \leq d_g(u^*) < k \cdot 2^{-n}.$$

Therefore, for all $u \in (-\infty, u^* + \delta) \cap \mathcal{R}$,

$$d_g(u) < k \cdot 2^{-n},$$

and so

$$u^* < u^* + \delta \le \inf \left\{ u \in \mathcal{R} : d_g(u) \ge k \cdot 2^{-n} \right\} = \ell(k, n).$$

In particular, it follows that

$$\left\{ u \in \mathcal{R} : (k-1) \cdot 2^{-n} \le d_g(u) < k \cdot 2^{-n} \right\} = [\ell(k-1, n), \ell(k, n)) \cap \mathcal{R}.$$

(If $k = 1$, simply replace the half-open interval with the open interval $(-\infty, \ell(1, n))$.) Since $F_n(u)$ is exactly equal to $(k-1) \cdot 2^{-n}$ on the latter set, Eq. (16) follows.

Now, since $\ell(k, n) = \ell(2^m \cdot k, n+m)$, it also follows from the definition of $F_n(u)$ in Eq. (15) that for all $n_0, n_1 \in \mathbb{N}$ and $u \in \mathbb{R}$,

$$|F_{n_0}(u) - F_{n_1}(u)| < 2^{-\min(n_0, n_1)}.$$

That is, the sequence $(F_n(u))_{n \in \mathbb{N}}$ converges uniformly on all of $\mathbb{R}$. Since $F_n(u)$ is non-decreasing, bounded between zero and one, and right-continuous for all n, it follows that the pointwise limit has these properties as well. That is,

$$F_{H_g}(u) \stackrel{\text{def}}{=} \lim_{n \to \infty} F_n(u)$$

is non-decreasing, bounded between zero and one, and right-continuous. Therefore $F_{H_g}(u)$ is the CDF of a random variable H_g.

However, using Eq. (16), we also have that

$$\sup \left| F_{H_g}(u) - d_g(u) \right| = \sup \lim_{n \to \infty} |F_n(u) - d_g(u)| \le \sup \lim_{n \to \infty} 2^{-n} = 0,$$

where the suprema are taken over $u \in \mathcal{R}$. Therefore $F_{H_g}(u) = d_g(u)$ for all $u \in \mathcal{R}$. Since $\Pr(U \in \mathcal{R}) = 1$, it follows that $d_g(u) = F_{H_g}(u)$ U-a.s., i.e., $d_g(u)$ generates the distribution of H_g. □

In light of Lemma S3, we note two important points about risk decision curves. First, a risk-decision curve can potentially generate more than one distribution. In particular, CDFs are defined on all of $\mathbb{R}$, whereas risk-decision curves are only defined U-a.s. Therefore, if U is not supported on all of $\mathbb{R}$, it may be the case that there is not a unique distribution H_g satisfying

$$d_g(u) = \Pr(H_g \le u) \qquad U\text{-a.s.} \tag{17}$$

In particular, $d_g(u)$ does not determine the distribution of H_g on regions outside the support of U. The functions $f(\,\cdot\,)$ we consider in the proof of Theorem S2 are constant on regions outside the support of U, however, meaning that the distributions of $f(H_g)$ and $f(\tilde{H}_g)$ are nevertheless identical for distinct H_g and $\tilde{H}_g$ generated by $d_g(u)$. Consequently, the different distributions $d_g(u)$ generates are, for our purposes, interchangeable. For this reason, we will on occasion refer to "the" distribution generated by $d_g(u)$, despite the ambiguity. (In particular, it is enough in Theorem S2 that the risk-decision curves generate *any* pair of distributions satisfying the MLRP.)

Second, as noted in Eq. (12), a generated distribution H_g is not necessarily a.s. finite. Throughout, we do not assume that *any* random variable is a.s. finite unless explicitly mentioned, as was the case for D, G, and U in SI B above.

D. Stochastic Orderings. As noted in Section 1, some hypotheses on the risk distributions are needed in Theorem S2 to rule out scenarios like the one shown in Figure S1. These hypotheses take the form of stochastic ordering relations, which we review here. The simplest and most important stochastic ordering is stochastic dominance.

Definition S4 (Stochastic Dominance)**.** Let F_0 and F_1 be arbitrary CDFs. We say that F_0 (first-order) *stochastically dominates* F_1, written $F_0 \succeq_1 F_1$, if for any $t \in \mathbb{R}$, $F_0(t) \le F_1(t)$. For random variables X and Y, we write $X \succeq_1 Y$ if $F_X \succeq_1 F_Y$, i.e., if

$$\Pr(X \le t) \le \Pr(Y \le t) \qquad \text{for all } t \in \mathbb{R}. \tag{18}$$

We say that a pair of random variables or distributions is *stochastically ordered* if one stochastically dominates the other.

Stochastic dominance has the useful property that increasing functions of the dominating random variable have greater expectation.

Lemma S5. *$X \succeq_1 Y$ if and only if $\mathbb{E}[f(X)] \ge \mathbb{E}[f(Y)]$ for any non-decreasing $f(x)$ for which the expectations are well-defined.*

For proof of Lemma S5, see, e.g., Shaked and Shantikumar (1). (The statement in Shaked and Shantikumar implicitly assumes that X and Y are a.s. finite, but the proof does not use this fact.)

To prove Theorem S2, we will need the following strengthening of stochastic dominance.

Definition S6 (Monotone Likelihood Ratio Property)**.** Let F_0 and F_1 be arbitrary cumulative distribution functions. Let f_0 and f_1 be the Radon-Nikodym derivatives (i.e., densities) of F_0 and F_1 with respect to their sum measure μ.[‡] We say that F_0 and F_1 have the *monotone likelihood ratio property* (MLRP) with F_0 dominating, denoted $F_0 \succeq_{\text{lr}} F_1$, if

$$\frac{f_0(x)}{f_1(x)}$$

is non-decreasing μ-a.e., where, without loss of generality, we define the ratio to be ∞ when $f_1(x) = 0$. For random variables X and Y, we write $X \succeq_{\text{lr}} Y$ if $F_X \succeq_{\text{lr}} F_Y$.

The MLRP has many useful consequences and appears widely in the literature. In addition to implying stochastic dominance (see Theorem 1.C.1 in Shaked and Shantikumar, ref. 1, and below), the MLRP holds for many familiar parametric families used to model risk distributions. For example:

- For $X \sim \text{Beta}(\alpha_0, \beta_0)$ and $Y \sim \text{Beta}(\alpha_1, \beta_1)$, $X \succeq_{\text{lr}} Y$ if and only if $\alpha_0 \geq \alpha_1$ and $\beta_0 \leq \beta_1$;
- For $X \sim \mathcal{N}(\mu_0, \sigma_0^2)$ and $Y \sim \mathcal{N}(\mu_1, \sigma_1^2)$, $X \succeq_{\text{lr}} Y$ if and only if $\mu_0 \geq \mu_1$ and $\sigma_0 = \sigma_1$;
- For $X \sim \text{Gamma}(\alpha_0, \beta_0)$ and $Y \sim \text{Gamma}(\alpha_1, \beta_1)$, $X \succeq_{\text{lr}} Y$ if and only if $\alpha_0 \geq \alpha_1$ and $\beta_0 \leq \beta_1$;
- For $X \sim \text{Binom}(n, p_0)$ and $Y \sim \text{Binom}(n, p_1)$, $X \succeq_{\text{lr}} Y$ if and only if $p_0 \geq p_1$.

We omit the routine verification of these facts. In the case of beta, normal, and gamma distributions, the MLRP is also equivalent to the densities of the risk distributions intersecting once.

Lemma S7. *Suppose $X \sim \text{Beta}(\alpha_0, \beta_0)$ and $Y \sim \text{Beta}(\alpha_1, \beta_1)$ have different distributions. Then X and Y satisfy the MLRP if and only if $f_X(p) = f_Y(p)$ for a unique $p \in (0, 1)$.*

The proof for normal and gamma distributions is similar.

Proof. We prove the lemma in three steps: First, we show that the densities must cross at least once. Next, we show that if the densities intersect more than once, then the MLRP does *not* hold. Finally, we show that if the densities intersect exactly once, then the MLRP *does* hold. We argue using the density ratio

$$r(p) \stackrel{\text{def}}{=} \frac{f_X(p)}{f_Y(p)} = \frac{B(\alpha_1, \beta_1)}{B(\alpha_0, \beta_0)} \cdot p^{\alpha_0 - \alpha_1} \cdot (1-p)^{\beta_0 - \beta_1}. \qquad [19]$$

Note that, taking derivatives, $r(p)$ is strictly monotonic if $\alpha_0 - \alpha_1$ and $\beta_0 - \beta_1$ have opposite signs or one of them is zero. (They cannot both be zero since the densities differ by assumption.) If $\alpha_0 - \alpha_1$ and $\beta_0 - \beta_1$ are both negative or both positive, it follows from Eq. (19) that

$$\lim_{p \to 0} r(p) = \lim_{p \to 1} r(p) = \infty \qquad \text{or} \qquad \lim_{p \to 0} r(p) = \lim_{p \to 1} r(p) = 0, \qquad [20]$$

respectively. Therefore $r(p)$ is non-monotonic in this case. In particular, irrespective of the parameters, $r(p)$ is either non-monotonic or strictly monotonic.

We now show that the densities must cross at least once. That is, there must exist $p_0 < p_* < p_1$ such that $f_X(p_*) = f_Y(p_*)$ and

$$\text{sgn}(f_X(p_0) - f_Y(p_0)) = -\text{sgn}(f_X(p_1) - f_Y(p_1)) \neq 0.$$

If not, then, without loss of generality, $f_X(p) \leq f_Y(p)$ for all $p \in (0, 1)$, with strict inequality holding for some p. Thus, by continuity, it follows that $\int_0^1 f_Y(p) - f_X(p)\, dp > 0$. This contradicts the fact that

$$\int_0^1 f_Y(p) - f_X(p)\, dp = \int_0^1 f_Y(p)\, dp - \int_0^1 f_X(p)\, dp = 1 - 1 = 0.$$

Therefore, there must exist p_0 and p_1 such that $f_X(p_0) - f_Y(p_0)$ and $f_X(p_1) - f_Y(p_1)$ have opposite signs. By the intermediate value theorem, therefore, there exists some p_* between p_0 and p_1 such that $f_X(p_*) = f_Y(p_*)$, yielding a crossing.

Next, suppose that $f_X(q_i) = f_Y(q_i)$ for $0 < q_0 < q_1 < 1$, so that the densities intersect at least twice. Then

$$r(q_0) = \frac{f_X(q_0)}{f_Y(q_0)} = 1 = \frac{f_X(q_1)}{f_Y(q_1)} = r(q_1),$$

[‡] I.e., for any interval $(a, b]$,

$$\mu[(a, b]] = F_0(b) + F_1(b) - (F_0(a) + F_1(a))$$

with μ suitably extended to all Borel sets by the Carathéodory extension theorem. Both F_0 and F_1 are absolutely continuous with respect to μ.

so $r(p)$ is not strictly monotonic, and hence by the argument above is non-monotonic. Therefore the MLRP does not hold by definition.

Finally, suppose that there is exactly one intersection at p_*. This intersection must therefore be a crossing. Assume without loss of generality that $r(p_0) < 1 < r(p_1)$. Then, by continuity, $r(p) < 1$ for all $p \in (0, p_*)$ and $r(p) > 1$ for all $p \in (p_*, 1)$. It follows immediately that $\alpha_0 - \alpha_1$ and $\beta_0 - \beta_1$ cannot both be positive, since then by Eq. (20), $r(0) = r(1) = 0$, and so by the intermediate value theorem there would be $p > p_*$ such that $r(x) < 1$. Similarly, they cannot both be negative. Therefore $\alpha_0 - \alpha_1$ and $\beta_0 - \beta_1$ have opposite signs, or one of them is zero, meaning that $r(p)$ is monotonic, i.e., that the MLRP holds. □

The MLRP is also preserved by monotone transformations: If $X \succeq_{\mathrm{lr}} Y$, then $g(X) \succeq_{\mathrm{lr}} g(Y)$ for monotonically increasing functions $g(x)$; see Theorem 2.5 in Keilson and Sumita (4). Thus, the MLRP also holds, e.g., with the analogous statements for log-normal, log-gamma, and logit-normal distributions. Like stochastic dominance, the MLRP is preserved when taking sums of random variables, so long as the densities are log-concave; see Lemma 1.1 in Shanthikumar and Yao (5). For further examples of conditions under which the MLRP holds, see Section 1.C of Shaked and Shanthikumar (1). In summary, the range of parametric families for which the MLRP holds is considerable.

In addition, the MLRP has many different equivalent formulations, some of which we note below.

Lemma S8. *Let X and Y be arbitrary random variables. Let $Z \sim \mathrm{Bernoulli}(p)$ for $p > 0$ be independent of X and Y, and let $W \stackrel{def}{=} Z \cdot X + (1 - Z) \cdot Y$. Then, the following are equivalent:*

1. *Monotone Likelihood Ratio Property (MLRP): The distributions of X and Y have the MLRP, i.e., $X \succeq_{\mathrm{lr}} Y$;*

2. *Uniform Conditional Stochastic Dominance (UCSD): For all events E such that $\Pr(X \in E) > 0$ and $\Pr(Y \in E) > 0$,*
$$[X \mid E] \succeq_1 [Y \mid E],$$
i.e., the distribution of X conditional on E stochastically dominates the distribution of Y conditional on E.

3. *Klinostochastic Dominance (KSD):*[§]
$$\frac{\int_{\bar{\mathbb{R}}} \mathbf{1}(x \le t) \cdot h(x)\, dF_X(x)}{\int_{\bar{\mathbb{R}}} h(x)\, dF_X(x)} \le \frac{\int_{\bar{\mathbb{R}}} \mathbf{1}(y \le t) \cdot h(y)\, dF_Y(y)}{\int_{\bar{\mathbb{R}}} h(y)\, dF_Y(y)}, \qquad [21]$$
for all $t \in \mathbb{R}$ and any $h(x)$ such that $h(X)$ and $h(Y)$ are a.s. non-negative and such that
$$0 < \mathbb{E}[h(X)] < \infty, \qquad 0 < \mathbb{E}[h(Y)] < \infty.$$

4. *Monotone conditional probability (MCP): The conditional probability*
$$g(w) \stackrel{def}{=} \Pr(Z = 1 \mid W = w)$$
is monotone W-a.s.

Proof. The equivalence between the MLRP and UCSD was originally given as Theorems 1.1 and 1.3 in Whitt (7)—though, see the note to Theorem 8 in Ruschendorf (6). The equivalence between the MLRP and KSD was first shown in Theorems 7 and 8 in Ruschendorf (6). (As above, the statements in these references assume that X and Y are a.s. finite, but the proofs do not use this fact.)

We now show the equivalence between MCP and the MLRP. The proof closely follows the sketch of the equivalence for distributions with densities in the proof of Theorem 1 above. Let $p = \Pr(Z = 1)$. We begin by observing that if
$$\begin{aligned} 0 &< \Pr(W \in E) \\ &= p \cdot \Pr(W \in E \mid Z = 1) + (1 - p) \cdot \Pr(W \in E \mid Z = 0) \\ &= p \cdot \Pr(X \in E) + (1 - p) \cdot \Pr(Y \in E), \end{aligned}$$
then
$$0 < \Pr(X \in E) + \Pr(Y \in E)$$

[§] This is the $\mathbb{B}^1$-ordering in the notation of Ruschendorf (6).

and *vice versa*. Therefore, the sum measure μ and the measure induced by F_W are mutually absolutely continuous. Consequently, by the chain rule for Radon-Nikodym derivatives,

$$\frac{f_X}{f_Y} = \frac{\frac{dF_X}{d\mu}}{\frac{dF_Y}{d\mu}} = \frac{\frac{dF_X}{d\mu} \cdot \frac{d\mu}{dF_W}}{\frac{dF_Y}{d\mu} \cdot \frac{d\mu}{dF_W}} = \frac{\frac{dF_X}{dF_W}}{\frac{dF_Y}{dF_W}}. \qquad [22]$$

Therefore, it suffices to compute the likelihood ratio with respect to F_W.

Now, note that

$$F_W = p \cdot F_{W|Z=1} + (1-p) \cdot F_{W|Z=0} = p \cdot F_X + (1-p) \cdot F_Y.$$

In particular, it follows from the linearity of the Radon-Nikodym derivative and the fact that the Radon-Nikodym derivative of a measure with respect to itself is unity that

$$1 - p \cdot \frac{dF_X}{dF_W} = (1-p) \cdot \frac{dF_Y}{dF_W}. \qquad [23]$$

Next, consider the measure $\nu : E \mapsto \Pr(W \in E \mid Z = 1)$—i.e., the distribution of X—and observe that

$$p \cdot \nu[E] = \Pr(W \in E, Z = 1) = \mathbb{E}[\Pr(Z = 1, W \in E \mid W)] = \mathbb{E}[\Pr(Z = 1 \mid W) \cdot \mathbf{1}(W \in E)].$$

Here the first equality follows from the definition of conditional probability, the second from the law of iterated expectation, and the third from the $\sigma(W)$-measurability of $\mathbf{1}(W \in E)$. Now, by the Doob-Dynkin lemma, there exists a $\sigma(W)$-measurable function $g(w)$ such that $\Pr(Z = 1 \mid W) = g(W)$ a.s. Therefore, the previous expression equals

$$\mathbb{E}[g(W) \cdot \mathbf{1}(W \in E)] = \int_E g(w)\, dF_W(w),$$

where the last equality is the fundamental property of pushforward measures; see, e.g., Theorem 3.6.1 in Bogachev (8). In particular, since

$$p \cdot \Pr(W \in E \mid Z = 1) = \int_E p \cdot \frac{dF_{W|Z=1}}{dF_W}(w)\, dF_w = \int_E p \cdot \frac{dF_X}{dF_w}(w)\, dF_w,$$

by the uniqueness of the Radon-Nikodym derivative,

$$g(w) = \Pr(Z = 1 \mid W = w) = p \cdot \frac{dF_X}{dF_W}(w) \qquad W\text{-a.s.}$$

Consider the function $h(q) : [0,1] \to [0, \infty]$ given by

$$h(q) \overset{\text{def}}{=} \frac{1-p}{p} \cdot \frac{q}{1-q},$$

where, without loss of generality, we set $h(1) = \infty$. Now,

$$h \circ g = h\left(p \cdot \frac{dF_X}{dF_W}\right) = \frac{1-p}{p} \cdot \frac{p \cdot \frac{dF_X}{dF_W}}{1 - p \cdot \frac{dF_X}{dF_W}} = \frac{1-p}{p} \cdot \frac{p \cdot \frac{dF_X}{dF_W}}{(1-p) \cdot \frac{dF_Y}{dF_W}} = \frac{\frac{dF_X}{dF_W}}{\frac{dF_Y}{dF_W}},$$

which is the likelihood ratio. The second equality here follows from Eq. (23). However, since $h(q)$ is monotonically increasing, $h \circ g$ is non-decreasing if and only if $g(w)$ is. Therefore, by Eq. (22), MCP holds if and only if the MLRP holds. □

Of these characterizations, the most important for proving Theorem S2 is klinostochastic dominance (KSD). For instance, we see by taking $h(x) = 1$ in Eq. (21) that KSD (and hence the MLRP) implies stochastic dominance. KSD is closely related to the notion of tilted distributions. For a random variable X and an X-a.s. non-negative function $h(x)$ such that $\mathbb{E}[h(X)]$ is finite and positive, we denote by $h \circlearrowleft X$ or $h \circlearrowleft F_X$ the *tilt* of the distribution of X by the weight function h, i.e., the probability distribution with CDF

$$F_{h \circlearrowleft X}(t) = \frac{\int_{\bar{\mathbb{R}}} \mathbf{1}(x \le t) \cdot h(x)\, dF_X}{\int_{\bar{\mathbb{R}}} h(x)\, dF_X} = \frac{\mathbb{E}[\mathbf{1}(X \le t) \cdot h(X)]}{\mathbb{E}[h(X)]}. \qquad [24]$$

KSD simply means that the stochastic ordering of two distributions is invariant under tilts: By Lemma S8, $X \succeq_{\text{lr}} Y$ if and only if

$$h \circlearrowleft X \succeq_1 h \circlearrowleft Y$$

for any appropriate weight function $h(x)$ (i.e., $h(x)$ such that $h(X)$ and $h(Y)$ are a.s. non-negative and $\mathbb{E}[h(X)]$ and $\mathbb{E}[h(Y)]$ are finite and positive).

E. Proof of correctness of the robust outcome test. KSD is the key ingredient needed to prove our main result: Under appropriate ordering assumptions on the risk distributions and risk-decision curves, the robust outcome test is correct.

Proof. We prove the contrapositive. That is, assume instead that

$$\Pr(d_0(U) < d_1(U)) > 0 \qquad \text{or} \quad d_0(u) = d_1(u) \quad U\text{-a.s.} \tag{25}$$

Note that by our second ordering assumption, $d_0(u)$ and $d_1(u)$ generate distributions with the MLRP. Since the MLRP implies stochastic dominance, it follows that either $d_0(u) \le d_1(u)$ U-a.s., or $d_1(u) \le d_0(u)$ U-a.s. By Eq. (25), it follows that $d_0(u) \le d_1(u)$.

Using the fact that $d_0(u) \le d_1(u)$, as in the proof of Theorem 1, we will show that either:

1. The decision rate is at least as large for group $G = 1$ as for group $G = 0$, i.e.,

$$\Pr(D = 1 \mid G = 0) \le \Pr(D = 1 \mid G = 1); \tag{26}$$

2. The outcome rate is no bigger for group $G = 1$ than for group $G = 0$, i.e.,

$$\mathbb{E}[U \mid D = 1, G = 0] \ge \mathbb{E}[U \mid D = 1, G = 1]. \tag{27}$$

Toward that end, let $U_0 \sim U \mid G = 0$ and $U_1 \sim U \mid G = 1$ be the conditional risk distributions, and let $d_0(u)$ and $d_1(u)$ generate distributions H_0 and H_1 with the MLRP. Then, there are two possibilities, which we will treat separately: Either $U_1 \succeq_{\mathrm{lr}} U_0$, or $U_0 \succeq_{\mathrm{lr}} U_1$.

Case 1 ($U_1 \succeq_{\mathrm{lr}} U_0$)**.** Suppose first that $U_1 \succeq_{\mathrm{lr}} U_0$. Then, in particular, $U_1 \succeq_1 U_0$. Since $d_0(u) \le d_1(u)$, we have that

$$\begin{aligned} \Pr(D = 1 \mid G = 0) &= \mathbb{E}[d_0(U) \mid G = 0] \\ &\le \mathbb{E}[d_0(U) \mid G = 1] \\ &\le \mathbb{E}[d_1(U) \mid G = 1] \\ &= \Pr(D = 1 \mid G = 1), \end{aligned}$$

i.e., the decision rate is at least as high for group $G = 1$ as for group $G = 0$, which is Eq. (26). Here, the equalities follow from the law of iterated expectations and the definition of $d_g(u)$ in Eq. (11). The first inequality follows from stochastic dominance and the fact that $d_0(u)$ is non-decreasing—because it exhibits bounded rationality—and the second inequality from the assumption that $d_0(u) \le d_1(u)$.

Case 2 ($U_0 \succeq_{\mathrm{lr}} U_1$)**.** Next, suppose that $U_0 \succeq_{\mathrm{lr}} U_1$. The proof in this case is similar, although more delicate. We will use KSD to show that for a fixed risk-decision curve, the outcome rate is higher for group $G = 0$ than group $G = 1$, and then we will use KSD again in a different way to show that for a fixed group $G = g$, the risk-decision curve $d_0(u)$ results in a higher outcome rate than the risk-decision curve $d_1(u)$.

In particular, we will need to consider the distributions $d_{g'} \circlearrowleft U_g$ for all $g, g' \in \{0, 1\}$, so we begin by verifying that these distributions are well-defined and have finite expectations.

Well-definedness and finite expectation of $d_{g'} \circlearrowleft U_g$. Let $d(u)$ be any risk-decision curve, i.e., a U-a.s. non-decreasing function taking values in $[0, 1]$. Then $\mathbb{E}[d(U) \mid G = g]$ is the corresponding decision rate for group $G = g$. In order for $d \circlearrowleft U_g$ to be well-defined, the definition of a tilted distribution in Eq. (24) requires that

$$0 < \int_{\mathbb{R}} d(u)\, dF_{U_g} = \mathbb{E}[d(u) \mid G = g] < \infty,$$

i.e., that the decision rate is positive. (For avoidance of doubt, by $\int \cdots\, dF_X$, we mean throughout the integral taken with respect to the pushforward measure $E \mapsto \Pr(X \in E)$ on $\bar{\mathbb{R}}$, not the Riemann-Stieltjes integral with respect to the integrator F_X.)

Finiteness follows immediately from the fact that $0 \le d(u) \le 1$ U-a.s. The positivity of $\mathbb{E}[d(U) \mid G = g]$ follows from our assumptions when $d(u) = d_{g'}(u)$. To see this, note that when $g = g'$, $\mathbb{E}[d_{g'}(U) \mid G = g] > 0$ by the non-triviality assumption in Eq. (8). On the other hand, when $g \ne g'$, observe that the overlap assumption in Eq. (10) implies that U_0 and U_1 are mutually absolutely continuous. Since $\mathbb{E}[d_{g'}(U) \mid G = g'] > 0$, there must exist some set E and $\epsilon > 0$ such that $d_{g'}(u) > \epsilon$ for all $u \in E$ and $\Pr(U \in E \mid G = g') > 0$. Therefore, in particular,

$$\mathbb{E}[d_{g'}(U) \mid G = g] \ge \epsilon \cdot \Pr(U \in E \mid G = g) > 0. \tag{28}$$

Here, the first inequality follows from the fact that $d_g(u) \geq \epsilon \cdot \mathbf{1}(u \in E)$. The second follows from mutual absolute continuity and the fact that $\Pr(U \in E \mid G = g') > 0$. In short, the tilted distributions $d_{g'} \circlearrowleft U_g$ are well-defined for all $g, g' \in \{0, 1\}$.

Next, we verify that the expectations of the tilted distributions $d_{g'} \circlearrowleft U_g$ are all finite. These expectations are the outcome rates of the risk-decision curve $d_{g'}(u)$ over the conditional risk distribution of group $G = g$ for all $g, g' \in \{0, 1\}$. Let $d(u)$ be an arbitrary risk-decision curve, and assume that the corresponding decision rate $\mathbb{E}[d(u) \mid G = g]$ is positive. Then, the absolute value of the outcome rate for group $G = g$ can be bounded as follows:

$$\left| \frac{\int_{\mathbb{R}} u \cdot d(u)\, dF_{U_g}}{\int_{\mathbb{R}} d(u)\, dF_{U_g}} \right| \leq \frac{\int_{\mathbb{R}} |u|\, dF_{U_g}}{\int_{\mathbb{R}} d(u)\, dF_{U_g}} = \frac{\mathbb{E}[|U| \mid G = g]}{\mathbb{E}[d(U) \mid G = g]} \leq \frac{\mathbb{E}[|U|]}{\Pr(G = g) \cdot \mathbb{E}[d(U) \mid G = g]}. \qquad [29]$$

Here the first inequality follows from the fact that $0 \leq d(u) \leq 1$ U-a.s. combined with the triangle inequality for integrals. The second inequality follows from the fact that for any non-negative random variable X and positive probability event E, $\mathbb{E}[X \mid E] \leq \mathbb{E}[X] \,/\, \Pr(E)$. We note that $\mathbb{E}[|U|]$ is finite by the assumption in Eq. (9) and $\Pr(G = g) > 0$ by the assumption in Eq. (7). In particular, when $d(u) = d_{g'}(u)$, $\mathbb{E}[d(U) \mid G = g]$ is positive by Eq. (28). Therefore the outcome rates are finite.

Comparison of outcome rates for $d_0(u)$ and $d_1(u)$. Having verified that the decision rates are positive and the outcome rates are finite for groups $G = 0$ and $G = 1$ under both risk-decision curves $d_0(u)$ and $d_1(u)$, we move on to comparing these rates.

Since $U_0 \succeq_{\mathrm{lr}} U_1$, we have by KSD in Lemma S8 that $d_1 \circlearrowleft U_0 \succeq_1 d_1 \circlearrowleft U_1$. It follows from Lemma S5 that the former outcome rate is greater than or equal to the latter outcome rate. To see this, note that $d_1(u)$ exhibits bounded rationality and is therefore non-decreasing. Additionally, the outcome rate

$$\frac{\int_{\mathbb{R}} u \cdot d_1(u)\, dF_{U_g}}{\int_{\mathbb{R}} d_1(u)\, dF_{U_g}}$$

is finite for $g \in \{0, 1\}$ by Eq. (29). Applying Lemma S5, we therefore have that

$$\mathbb{E}[U \mid D = 1, G = 1] = \frac{\int_{\mathbb{R}} u \cdot d_1(u)\, dF_{U_1}}{\int_{\mathbb{R}} d_1(u)\, dF_{U_1}} \leq \frac{\int_{\mathbb{R}} u \cdot d_1(u)\, dF_{U_0}}{\int_{\mathbb{R}} d_1(u)\, dF_{U_0}}. \qquad [30]$$

Next, we show that switching the risk-decision curve from $d_1(u)$ to $d_0(u)$ can only increase the outcome rate. To see this, we proceed in four steps. First, note that by the definition of H_g, we have that

$$\frac{\int_{\mathbb{R}} u \cdot d_g(u)\, dF_{U_0}(u)}{\int_{\mathbb{R}} d_g(u)\, dF_{U_0}(u)} = \frac{\int_{\mathbb{R}} u \cdot \left[\int_{\bar{\mathbb{R}}} \mathbf{1}(s \leq u)\, dF_{H_g}(s)\right] dF_{U_0}(u)}{\int_{\mathbb{R}} \left[\int_{\bar{\mathbb{R}}} \mathbf{1}(s \leq u)\, dF_{H_g}(s)\right] dF_{U_0}(u)}.$$

Second, by Eq. (29), we can apply the Fubini-Tonelli theorem to the latter expression, yielding

$$\frac{\int_{\mathbb{R}} u \cdot d_g(u)\, dF_{U_0}(u)}{\int_{\mathbb{R}} d_g(u)\, dF_{U_0}(u)} = \frac{\int_{\bar{\mathbb{R}}} \left[\int_{\mathbb{R}} u \cdot \mathbf{1}(u \geq s)\, dF_{U_0}(u)\right] dF_{H_g}(s)}{\int_{\bar{\mathbb{R}}} \left[\int_{\mathbb{R}} \mathbf{1}(u \geq s)\, dF_{U_0}(u)\right] dF_{H_g}(s)}.$$

Third, multiplying and dividing the outer integrand in the numerator on the right-hand side by $\int_{\mathbb{R}} \mathbf{1}(u \geq s)\, dF_{U_0}(u)$ (and, when $\Pr(U \geq s \mid G = 0) = 0$, defining this ratio without loss of generality to be zero) yields that

$$\frac{\int_{\mathbb{R}} u \cdot d_g(u)\, dF_{U_0}(u)}{\int_{\mathbb{R}} d_g(u)\, dF_{U_0}(u)} = \frac{\displaystyle\int_{\bar{\mathbb{R}}} \left[\frac{\int_{\mathbb{R}} u \cdot \mathbf{1}(u \geq s)\, dF_{U_0}(u)}{\int_{\mathbb{R}} \mathbf{1}(u \geq s)\, dF_{U_0}(u)}\right] \cdot \left[\int_{\mathbb{R}} \mathbf{1}(u \geq s)\, dF_{U_0}(u)\right] dF_{H_g}(s)}{\int_{\bar{\mathbb{R}}} \left[\int_{\mathbb{R}} \mathbf{1}(u \geq s)\, dF_{U_0}(u)\right] dF_{H_g}(s)}. \qquad [31]$$

Fourth and finally, from the form of the integral, we see that the right-hand side of Eq. (31) is an expectation. In particular, it is the expectation of a non-decreasing function $f(s)$:

$$f(s) \stackrel{\text{def}}{=} \frac{\int_{\mathbb{R}} u \cdot \mathbf{1}(u \geq s)\, dF_{U_0}(u)}{\int_{\mathbb{R}} \mathbf{1}(u \geq s)\, dF_{U_0}(u)} = \mathbb{E}[U \mid U \geq s, G = 0].$$

The expectation in Eq. (31) is taken with respect to the distribution $h \circlearrowleft H_g$, where

$$h(s) \stackrel{\text{def}}{=} \int_{\mathbb{R}} \mathbf{1}(u \geq s)\, dF_{U_0}(u) = \Pr(U \geq s \mid G = 0).$$

(Since $h(s) > 0$ if and only if $\Pr(U \geq s \mid G = 0) > 0$, $f(s)$ is well-defined $h \circlearrowleft H_g$-a.s.) By Eq. (31) the expectation of $f(s)$ with respect to the distribution $h \circlearrowleft H_g$ equals

$$\frac{\int_{\mathbb{R}} u \cdot d_g(u)\, dF_{U_0}(u)}{\int_{\mathbb{R}} d_g(u)\, dF_{U_0}(u)},$$

and so the expectation is consequently finite by Eq. (29).

Putting these pieces together, since $H_0 \succeq_{\mathrm{lr}} H_1$ by hypothesis, it follows that $h \circlearrowleft H_0 \succeq_1 h \circlearrowleft H_1$. Thus, since $f(s)$ is non-decreasing and has finite expectation, it follows by Lemma S5 that its expectation with respect to $h \circlearrowleft H_0$ is at least as large as its expectation with respect to $h \circlearrowleft H_1$. In other words, the expression in Eq. (31) with $g = 0$ is greater than or equal to the expression with $g = 1$, i.e.,

$$\frac{\int_{\mathbb{R}} u \cdot d_1(u)\, dF_{U_0}}{\int_{\mathbb{R}} d_1(u)\, dF_{U_0}} \leq \frac{\int_{\mathbb{R}} u \cdot d_0(u)\, dF_{U_0}}{\int_{\mathbb{R}} d_0(u)\, dF_{U_0}} = \mathbb{E}[U \mid D = 1, G = 0].$$

Combining this with Eq. (30) gives that the outcome rate is at least as small for group $G = 1$ as group $G = 0$, i.e.,

$$\mathbb{E}[U \mid D = 1, G = 1] \leq \mathbb{E}[U \mid D = 1, G = 0],$$

which is Eq. (27).

□

F. A Note on Sampling Error. To clarify our main conceptual points, we have focused throughout on the infinite data regime, where decision and outcome rates are estimated with negligible error. In practice, however, sampling error can be an important concern.

For notational simplicity, let DR_g and OR_g denote the true decision and outcome rates for group g—i.e., $\Pr(D = 1 \mid G = g)$ and $\mathbb{E}[U \mid D = 1, G = g]$. Let

$$n_g \stackrel{\text{def}}{=} \sum_{i=1}^{n} \mathbf{1}(G_i = g) \qquad \text{and} \qquad n_{g,d} \stackrel{\text{def}}{=} \sum_{i=1}^{n} \mathbf{1}(G_i = g, D_i = d)$$

denote the number of individuals in group $G = g$ and, respectively, the number of observations both in group $G = g$ and receiving decision $D = d$. Consistent with our notation in Section 2.B, let $\widehat{\mathrm{DR}}_g$ and $\widehat{\mathrm{OR}}_g$ for $g \in \{0, 1\}$ denote the empirical decision and outcome rates, i.e.,

$$\widehat{\mathrm{DR}}_g \stackrel{\text{def}}{=} \frac{n_{g,1}}{n_g}, \qquad \widehat{\mathrm{OR}}_g \stackrel{\text{def}}{=} \frac{\sum_{i=1}^{n} U_i \cdot \mathbf{1}(G_i = g, D_i = 1)}{n_{g,1}}.$$

Set

$$\widehat{\mathrm{SVar}}_{U,g} \stackrel{\text{def}}{=} \frac{\sum_{i=1}^{n} \mathbf{1}(G_i = g, D_i = 1) \cdot (U_i - \widehat{\mathrm{OR}}_g)^2}{n_g - 1}.$$

Finally, let $\Delta_{\mathrm{DR}} \stackrel{\text{def}}{=} \mathrm{DR}_1 - \mathrm{DR}_0$ denote the true difference in decision rates, with Δ_{OR}, $\hat{\Delta}_{\mathrm{DR}}$, and $\hat{\Delta}_{\mathrm{OR}}$ defined analogously.

To quantify uncertainty in $\hat{\Delta}_{\mathrm{DR}}$ and $\hat{\Delta}_{\mathrm{OR}}$, first observe that $\widehat{\mathrm{DR}}_0$, $\widehat{\mathrm{DR}}_1$, $\widehat{\mathrm{OR}}_0$, and $\widehat{\mathrm{OR}}_1$ are approximately independent: Dependency arises only because n_0, n_1, $n_{0,1}$, and $n_{1,1}$ are not fixed.[¶] As a result, we can estimate the standard errors of $\hat{\Delta}_{\mathrm{DR}}$ and $\hat{\Delta}_{\mathrm{OR}}$ as follows:

$$\widehat{\mathrm{SErr}}_{\Delta_{\mathrm{DR}}} \stackrel{\text{def}}{=} \sqrt{\frac{\widehat{\mathrm{DR}}_0 \cdot \left(1 - \widehat{\mathrm{DR}}_0\right)}{n_0} + \frac{\widehat{\mathrm{DR}}_1 \cdot \left(1 - \widehat{\mathrm{DR}}_1\right)}{n_1}}, \qquad \widehat{\mathrm{SErr}}_{\Delta_{\mathrm{OR}}} \stackrel{\text{def}}{=} \sqrt{\frac{\widehat{\mathrm{SVar}}_{U,0}}{n_{0,1}} + \frac{\widehat{\mathrm{SVar}}_{U,1}}{n_{1,1}}}.$$

From this approximate independence, it also follows immediately that the most compact central confidence region is given by

$$C\left(\hat{\Delta}_{\mathrm{DR}}, \hat{\Delta}_{\mathrm{OR}}; \alpha\right) \stackrel{\text{def}}{=} \left\{ (\Delta_{\mathrm{DR}}, \Delta_{\mathrm{OR}}) : \left(\frac{\Delta_{\mathrm{DR}} - \hat{\Delta}_{\mathrm{DR}}}{\widehat{\mathrm{SErr}}_{\Delta_{\mathrm{DR}}}}\right)^2 + \left(\frac{\Delta_{\mathrm{OR}} - \hat{\Delta}_{\mathrm{OR}}}{\widehat{\mathrm{SErr}}_{\Delta_{\mathrm{OR}}}}\right)^2 \leq x_{1-\alpha} \right\}, \qquad [32]$$

where $x_{1-\alpha}$ is the $(1-\alpha)$-quantile of the $\chi^2(2)$ distribution. Equivalently, $C\left(\hat{\Delta}_{\mathrm{DR}}, \hat{\Delta}_{\mathrm{OR}}; \alpha\right)$ corresponds to thresholding the joint density of independent $\mathcal{N}\left(\hat{\Delta}_{\mathrm{DR}}, \widehat{\mathrm{SErr}}_{\Delta_{\mathrm{DR}}}\right)$ and $\mathcal{N}\left(\hat{\Delta}_{\mathrm{OR}}, \widehat{\mathrm{SErr}}_{\Delta_{\mathrm{OR}}}\right)$

[¶] In particular, using the delta method and central limit theorem, it is straightforward to show that as $n \to \infty$, $(\hat{\Delta}_{\mathrm{DR}} - \Delta_{\mathrm{DR}}) \,/\, \widehat{\mathrm{SErr}}_{\Delta_{\mathrm{DR}}}$ and $(\hat{\Delta}_{\mathrm{OR}} - \Delta_{\mathrm{OR}}) \,/\, \widehat{\mathrm{SErr}}_{\Delta_{\mathrm{OR}}}$ converge in distribution to independent standard normals.

distributions above an appropriate threshold t_α to obtain the region of highest density. This results in an elliptical confidence region for Δ_{DR} and Δ_{OR} centered at $\left(\hat{\Delta}_{\text{DR}}, \hat{\Delta}_{\text{OR}}\right)$, with the major and minor axes determined by the estimated standard errors of the sample difference in decision and outcome rates. Asymptotically valid hypothesis tests can be straightforwardly obtained by, for example, taking the maximum p-value of one-tailed tests of $\Delta_{\text{DR}} \leq 0$ and $\Delta_{\text{OR}} \geq 0$.

2. Data and Risk Estimation

Our empirical results are based on data drawn from five sources:

- **Police Stops**: Administrative data gathered under the California Racial Identity and Profiling Act (9);
- **Lending**: Lending data drawn from applicants to a large online financial technology platform;
- **Recidivism**: COMPAS recidivism prediction scores from Broward County, Florida (10);
- **Contraband**: Administrative data from the New York Police Department's Stop, Question, and Frisk program (11);
- **Bar passage**: Law school admissions and graduation data from the Law School Admissions Council's Longitudinal Bar Passage Study (12).

Data sources, pre-processing steps, risk model specifications, exclusion criteria, and other details are given below.

A. Police Stops. The police stops dataset used in this analysis is drawn from data gathered in 2022 pursuant to California's Racial Identity and Profiling Act (RIPA). Under RIPA, law enforcement officers must record a wide range of information about stops they make of vehicles and pedestrians, including the stop circumstances (e.g., date and time), demographics of the stopped individual (e.g., race and age), reasons for the stop (e.g., traffic violation or matching the description of a person of interest), outcomes of the stop (e.g., weapons or other contraband found), actions taken during the stop (e.g., arrest or use of force), and other information. First enacted in 2015, RIPA requirements were extended to all law enforcement agencies in the state in 2022.

The full dataset comprises more than 4.5 million records of police stops from 536 law enforcement agencies (9). Following Grossman et al. (13), we restrict our analysis to non-consensual, discretionary stops not conducted in an educational context. That is, we exclude:

1. Stops undertaken because the officer had knowledge of an arrest warrant or some form of mandatory supervision (i.e., probation, parole, or post-release community supervision);
2. Consensual encounters with motorists or pedestrians, which are recorded inconsistently across agencies;
3. Stops that occurred in an educational context, or for education-related reasons, such as truancy, as the threshold for conducting stops of this nature can be lower than for other types of stops. (See, e.g., *The People v. William G.*, ref. 14.)

To ensure that sufficient data exist to accurately estimate the decision and outcome rates, we further restrict our analysis to agencies that conducted at least 1,000 stops of Black, Hispanic, and White individuals in 2022. This leaves us with a final dataset of approximately 2.8 million stops from 56 law enforcement agencies.

B. Lending.

Data Source Our lending data come from an online financial technology platform that facilitates lending to individuals for a variety of purposes based on a proprietary underwriting model that utilizes both traditional and non-traditional information to assess lending risk. Our training data consist of approximately 300,000 borrowers who applied for loans between January 2019 and July 2021 for whom repayment outcomes are known. While race is not recorded in the training data, it is imputed using names and geographic information using Bayesian Improved Surname Geocoding or "BISG" (15). For each individual, we impute the most likely race using the BISG model, and then subset to individuals whose imputed race is Black, Hispanic, or White. We then fit our model on the approximately 260,000 individuals remaining. To understand the distribution of risk among loan applicants, we apply our fitted risk model to a separate set of 130,000 individuals who applied for loans in early- to mid-August 2021, but who did not necessarily receive a loan.

Risk Model We fit a gradient-boosted decision tree model to predict the likelihood that an individual will default before the end of the loan term using a bevy of traditional and non-traditional covariates, including credit score, available at the time of their application. The model achieves an AUC of 73% on a 10% set of held-out training data, and is well-calibrated, as shown in Figure S2.

C. Recidivism.

Data Source The recidivism dataset used in this analysis comes from ProPublica's analysis of COMPAS recidivism prediction scores from Broward County, Florida (10). The dataset contains COMPAS scores for approximately 12,000 individuals who were assessed for recidivism risk pre-trial in 2013 and 2014, as well as information on whether they were rearrested within two years of their initial assessment. After subsetting to the collection of individuals for whom outcomes are known, and whose race is recorded as Black, Hispanic, or White, we are left with a final dataset of approximately 11,000 observations.

Risk Model To predict probability of recidivism, we use raw COMPAS scores, which range continuously from approximately -3 to 3. To obtain calibrated probabilities, we fit a logistic regression model to predict recidivism from the interaction of the raw COMPAS score and race. The model achieves an AUC of 68% on the held-out training data, which is consistent with the AUC reported for COMPAS scores in a variety of jurisdictions (10, 16–24). The model is also well-calibrated, as shown in Figure S2.

D. Contraband.

Data Source Under *Terry v. Ohio*, Police officers may stop and question pedestrians about whom they have "reasonable suspicion" of criminal activity, and may conduct a "frisk" of the individual's outer clothing if they believe the stopped individual is in possession of a weapon (25). Between 2003 and 2013, the New York Police Department (NYPD) conducted over 100,000 such *Terry* stops per year. Officers recorded details of these stops, including stop circumstances (e.g., time and location), reasons for suspicion (e.g., suspicious bulge or furtive movements), stop outcomes (e.g., arrest or contraband found), and demographic information about the stopped individual. We use records of the approximately 2.7 million stops conducted between 2008 and 2013. Our training set consists of the approximately 800,000 stops conducted between 2008 and 2010 in which the stopped individual was frisked, and consequently for which it is known whether or not the individual was in possession of a weapon. We split the training data into an 80% training set, which we use to fit our model predicting weapon possession, and a 20% holdout set, which we use to evaluate the performance of our model. To understand the overall distribution of risk among stopped individuals, we apply our fitted risk model to the approximately 1.1 million stops conducted in 2011 and 2012.

Risk Model We fit a gradient-boosted decision tree model to predict the likelihood that an individual who is frisked will be found to be in possession of a weapon using 33 covariates known at the time of the stop and before the frisk decision is made. These covariates include the time and location of the stop, the officer's reason for making the stop, and additional circumstances related to the stop, such as whether the individual was in proximity to a crime scene. The model achieves an AUC of 79% on the held-out training data. The model is reasonably well-calibrated for risks below 9% (approximately the 99th percentile of the risk distribution), although it is modestly over-predictive of risk for White individuals; see Figure S2.

E. Admissions.

Data Source We draw our bar passage dataset from the Law School Admissions Council's Longitudinal Bar Passage Study (12), which studied bar passage rates among 163 of 172 ABA-accredited law schools in the United States. Study participants represent around 23,000 out of the approximately 40,000 law school students who entered law school in the fall of 1991. The dataset includes information about the law schools attended by the students, their undergraduate GPAs, their LSAT scores, their gender and race, and whether they passed the bar exam on their first or second attempt.

Risk Model We fit a logistic regression model to predict the likelihood that an individual eventually passed the bar exam using the individual's LSAT score, undergraduate GPA, a measure of socioeconomic status, gender, and race. The model achieves an AUC of 79%, and is well-calibrated, as shown in Figure S2: For Black and Hispanic applicants, the model is well-calibrated across a range of estimated risks, and for White applicants, it is well-calibrated for risks greater than roughly 90% (approximately the 3rd percentile of risk for White applicants).

3. Simulation Study

A. Violations of the MLRP. We present the results of the simulation study described in the main text for Hispanic individuals in Figure S3. We further extend our simulation study to quasi-rational decision policies of the following form:

$$d_g(r) = F(r; t_g, \sigma) \stackrel{\text{def}}{=} \Phi\left(\frac{\text{logit}^{-1}(r) - \text{logit}^{-1}(t_g)}{\sigma}\right), \qquad [33]$$

where $\Phi(x)$ is the standard normal CDF. Here, $F(x; t, \sigma)$ is the cumulative distribution function of the logit-normal distribution with center t (on the probability scale) and scale parameter σ. To model a realistic level of bounded rationality, we set σ to be one half of the standard deviation of the overall distribution of estimated risk on the logit scale. (In particular, we set σ to be 0.62 logits for the lending dataset, 0.36 logits for the recidivism dataset, 0.45 logits for the contraband dataset, and 0.51 logits for the admissions dataset.) As before, we then sweep over all percentiles t_g (excluding the 0th and 100th percentiles); see Figure S4 for examples of the quasi-rational risk-decision curves used in the simulation, where t_g is set to the 1/3, 1/2, and 2/3 quantiles, respectively. At each percentile, we estimate the decision and outcome rates for group $G = g$ as follows:[‖]

$$\widehat{\text{DR}}_g \stackrel{\text{def}}{=} \frac{1}{n_g}\sum_{i=1}^{n} \mathbf{1}(G = g) \cdot d_g(\hat{R}_i), \qquad \widehat{\text{OR}}_g \stackrel{\text{def}}{=} \frac{\sum_{i=1}^{n} \mathbf{1}(G = g) \cdot d_g(\hat{R}_i) \cdot \hat{R}_i}{\sum_{i=1}^{n} \mathbf{1}(G = g) \cdot d_g(\hat{R}_i)}, \qquad [34]$$

where $d_g(r)$ is the risk-decision curve being tested for group $G = g$, $\hat{R}_i$ is the estimated risk for individual i, and n_g is the number of individuals in group g. (We note that this is equivalent to the method used in the main text, differing only in that the risk-decision curve takes values in $[0, 1]$.) We then estimate the difference in decision and outcome rates between groups g_1 and g_0 as

$$\widehat{\text{DR}}_{g_1} - \widehat{\text{DR}}_{g_0}, \qquad \widehat{\text{OR}}_{g_1} - \widehat{\text{OR}}_{g_0},$$

and test for discrimination using the robust and standard outcome tests accordingly.

Figures S5 and S6 show that broadly similar, if even more pronounced, results hold when the risk-decision curves are quasi-rational as opposed to fully rational. In particular, the robust outcome test rarely detects discrimination when decisions are made in the same way for both groups, and usually detects discrimination when there is a double standard. In particular, in cases where the base rates differ substantially between groups and the decision maker has limited sensitivity to risk, the standard outcome test can essentially always "detect" that there is a lower decision standard for the lower base rate group, regardless of the actual decision standards.

B. Differences in Base Rates. The range of cases in which the robust outcome test detects discrimination rather than returning an inconclusive result varies across our examples. In the simulations shown in Figures 3, S3, S5, and S6, the robust outcome test is inconclusive in more scenarios in examples where the base rates of the groups are more different.

To gauge whether differences in base rates reduce the robust outcome test's sensitivity more broadly, we additionally simulate a range of discriminatory and non-discriminatory scenarios where we allow base rates to differ to a greater or lesser extent. More precisely, we consider risk distributions of the form

$$R \mid G = g \sim \text{Beta}(\mu_g, \nu),$$

i.e., where the risk distribution for group $G = g$, $g \in \{0, 1\}$, is beta distributed with mean μ_g and total count parameter ν.[**] We consider all possible threshold decision policies with $t_0, t_1 \in \{1\%, 2\%, \ldots, 99\%\}$, and report the proportion of scenarios in which the robust outcome test is conclusive, i.e., indicates discrimination against one group or the other. We sweep over $\mu_0 \in \{10\%, 20\%, \ldots, 90\%\}$, $\mu_1 \in \{1\%, 2\%, \ldots, 99\%\}$, and $\nu \in \{1, 2, 4, \ldots, 64\}$. The results are shown in Figure S7. Across total count parameters and base rates μ_0 for group $G = 0$, a pattern consistent with our observations using empirical risk distributions holds: Increasing the gap between the high and low base rate groups results in an inconclusive robust outcome test more often.

[‖] Our notation in Eq. (34) differs slightly from Section 2.B and SI F to take advantage of the fact that the risk decision curves are known exactly.

[**] In the standard parameterization of the beta distribution, $\alpha = \mu \cdot \nu$ and $\beta = (1 - \mu) \cdot \nu$.

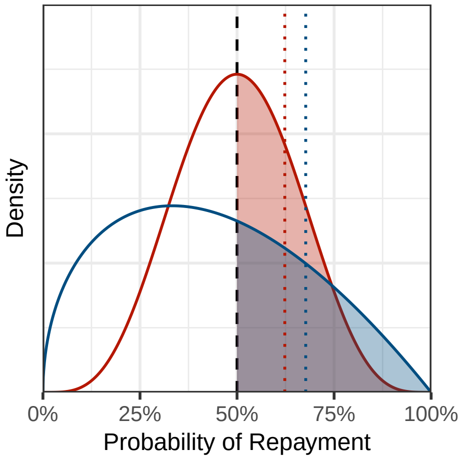

Fig. S1. *A stylized illustration of a pair of risk distributions and lending decisions for which the robust outcome test would be incorrect. The lending rates correspond to the areas of the colored regions (viz., 50% for the red group and 38% for the blue group), and the repayment rates are shown by the dotted red and blue lines (viz., 62% for the red group and 67% for the blue group). Applying a uniform lending threshold (50%) results in a lower lending rate to applicants from the blue group, as well as a higher repayment rate, leading the robust outcome test to erroneously conclude that the thresholds differ for the two groups.*

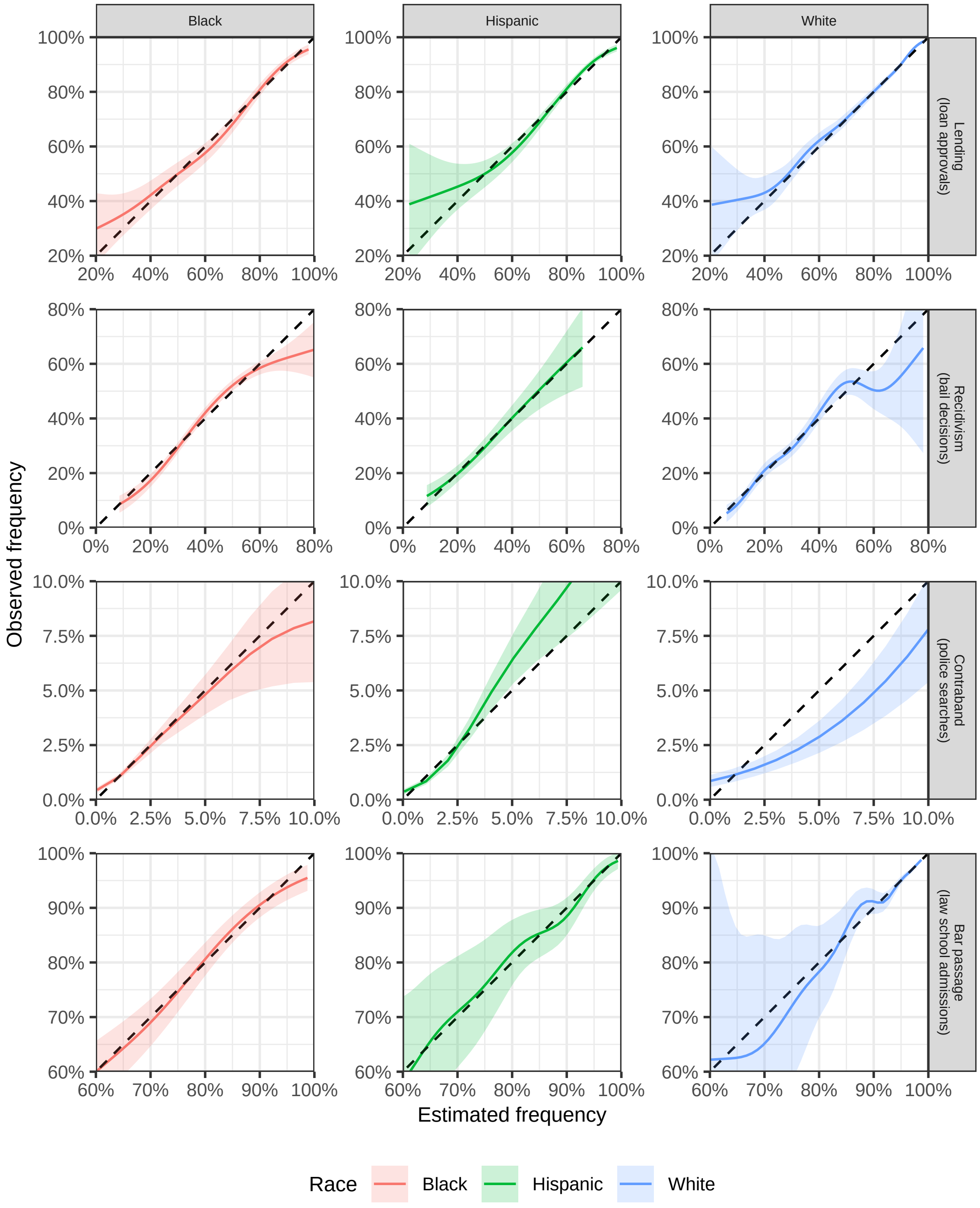

Fig. S2. *Calibration curves for the risk models used in our analysis. The x-axis indicates the estimated proportion of cases in which the predicted event (viz., loan repayment, recidivism, weapon possession, or bar passage) occurs, and the y-axis indicates the actual proportion of cases in which the event occurs. The diagonal line indicates perfect calibration. The smooth curves represent logistic regression GAM models fit to the data using a thin plate regression spline basis. The shaded regions indicate 95% confidence intervals.*

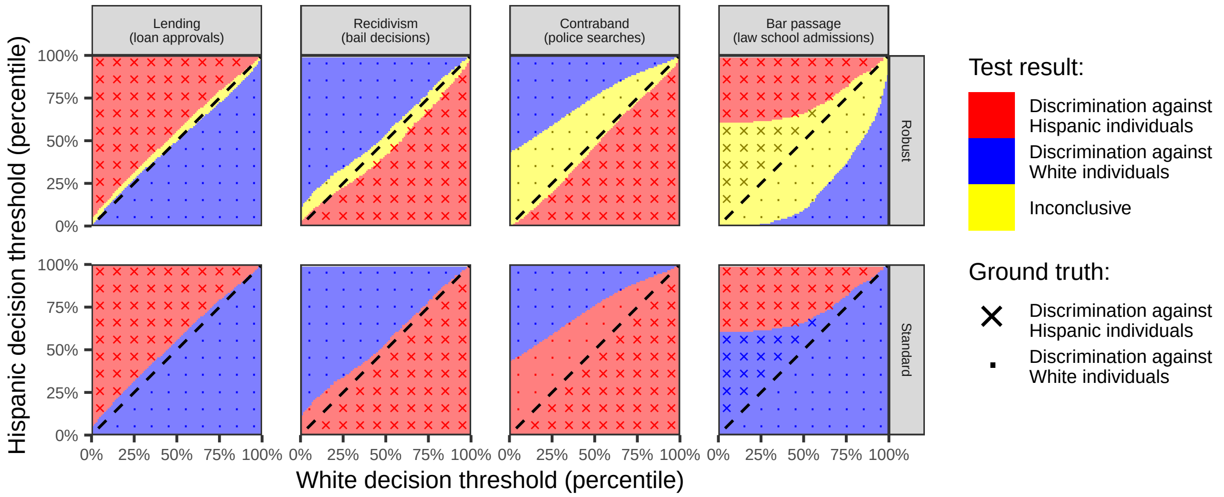

Fig. S3. *Results of a simulation study comparing the standard and robust outcome tests. The x-axis indicates the decision threshold for White individuals, and the y-axis indicates the threshold for Hispanic individuals. The upper-left and lower-right triangular regions correspond to scenarios where decision makers discriminate against either Hispanic or White individuals, as indicated by the "$\times$" and "$\cdot$" symbols, respectively; non-discriminatory scenarios are shown by the dashed diagonal line. Red regions indicate where the tests suggest discrimination against Hispanic individuals, blue regions indicate where the tests suggest discrimination against White individuals, and yellow regions indicate where the robust outcome test is inconclusive. The gray areas represent simulation scenarios that are not feasible because a threshold lies outside the support of the risk distribution of the corresponding group. Across simulations, the standard outcome test indicates discrimination when, in reality, there is none—and often indicates discrimination against the group that in actuality was favored. In contrast, the robust outcome test is nearly always directionally accurate, though it sometimes returns an inconclusive result.*

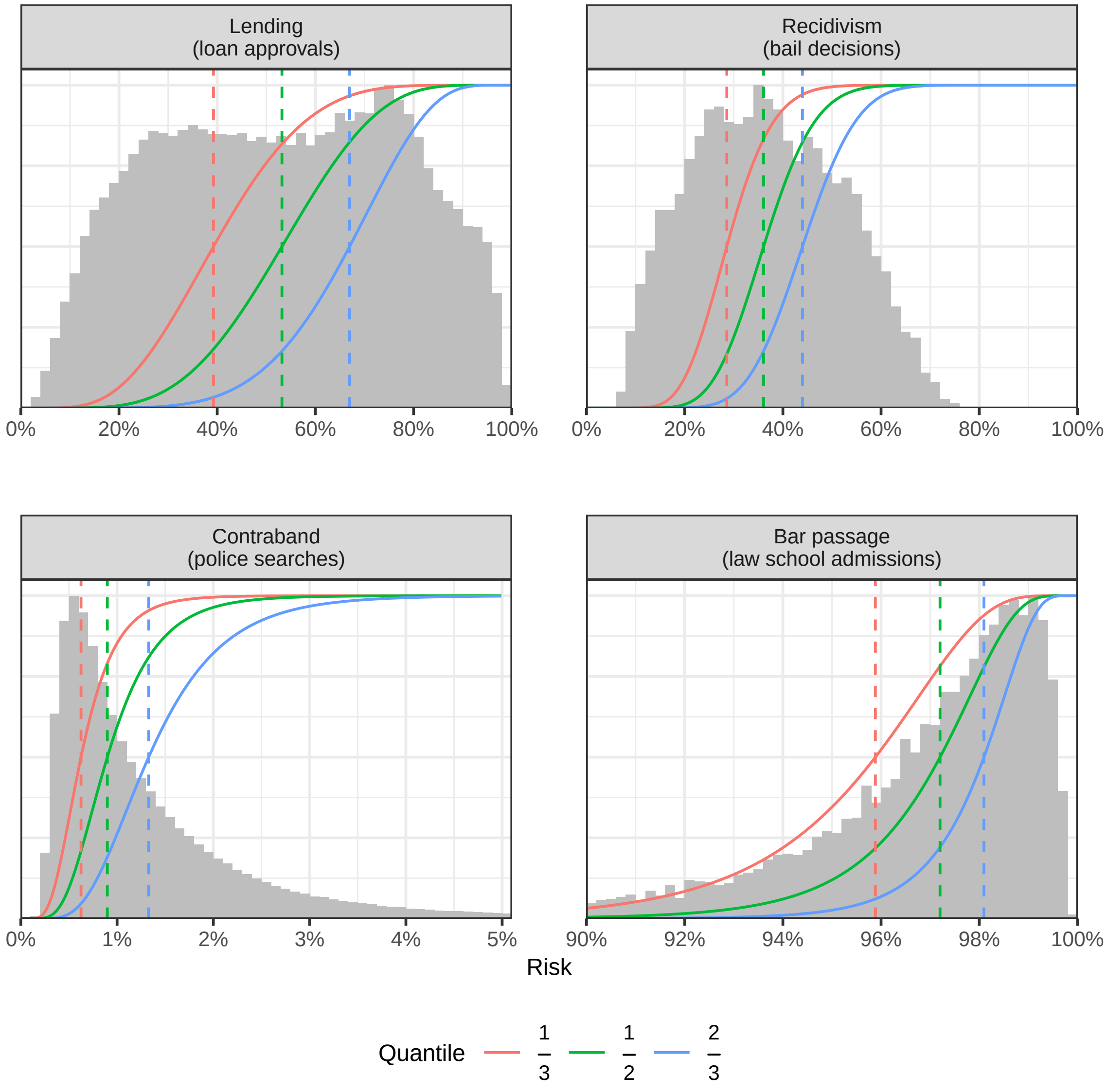

Fig. S4. *Examples of the quasi-rational risk-decision curves used in the simulation study. The x-axis indicates the estimated risk. The overall distribution of risk is shown by the gray histogram. The red, green, and blue curves show the quasi-rational risk-decision curves centered, respectively, at the $1/3$, $1/2$, and $2/3$ quantiles—indicated by red, green, and blue dashed vertical lines.*

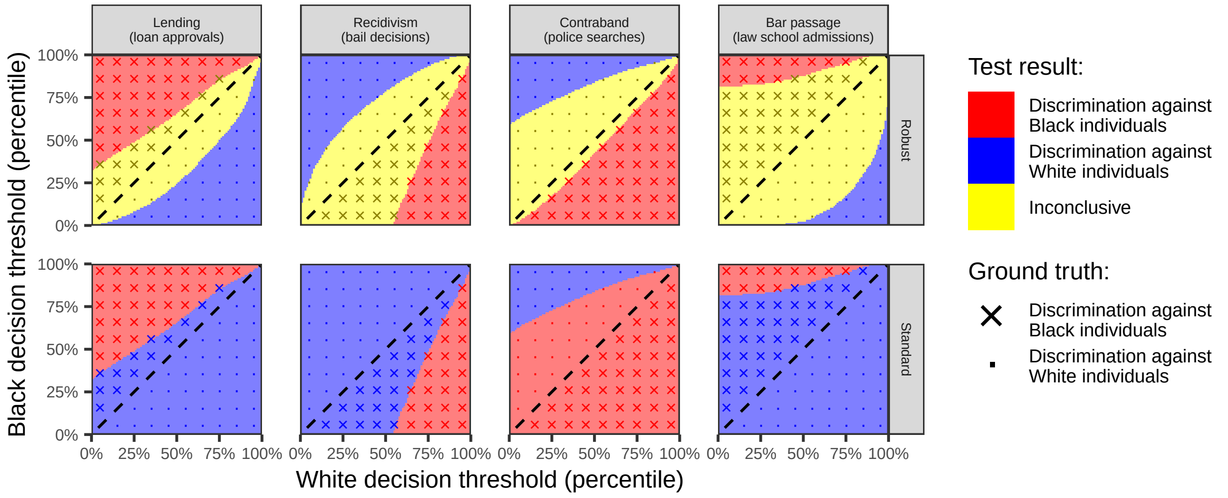

Fig. S5. *Results of a simulation study comparing the standard and robust outcome tests with quasi-rational (i.e., logit-normal CDF) risk-decision curves. The x-axis indicates the "center"—i.e., t, in Eq. (33)—for White individuals, and the y-axis indicates the center for Black individuals. The upper-left and lower-right triangular regions correspond to scenarios where decision makers discriminate against either Black or White individuals, as indicated by the "$\times$" and "$\cdot$" symbols, respectively; non-discriminatory scenarios are shown by the dashed diagonal line. Red regions indicate where the tests suggest discrimination against Black individuals, blue regions indicate where the tests suggest discrimination against White individuals, and yellow regions indicate where the robust outcome test is inconclusive. Across simulations, the standard outcome test indicates discrimination when, in reality, there is none—and often indicates discrimination against the group that in actuality was favored. In contrast, the robust outcome test is nearly always directionally accurate, though it sometimes returns an inconclusive result.*

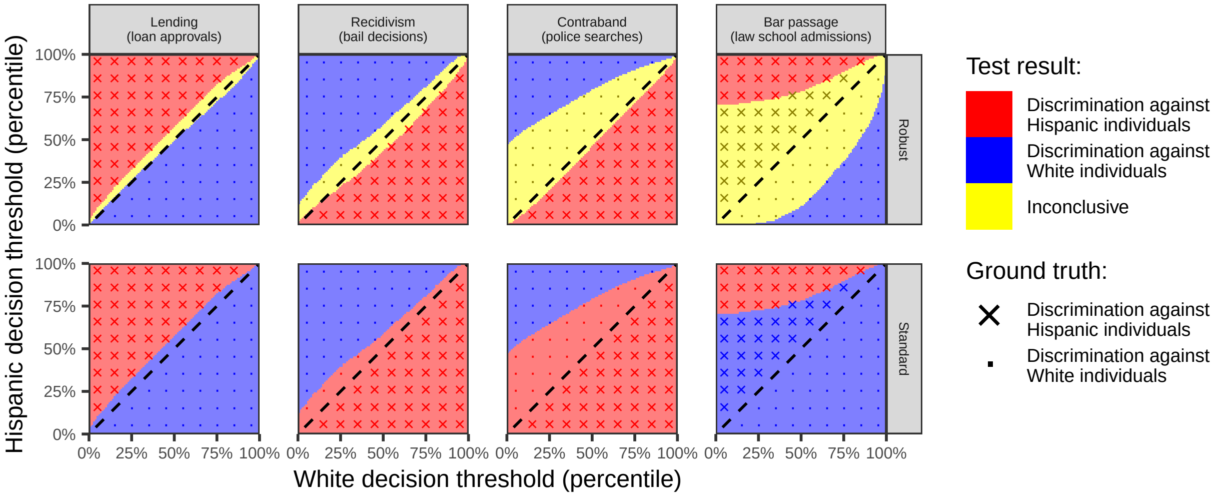

Fig. S6. *Results of a simulation study comparing the standard and robust outcome tests with quasi-rational (i.e., logit-normal CDF) risk-decision curves. The x-axis indicates the "center"—i.e., t, in Eq. (33)—for White individuals, and the y-axis indicates the center for Hispanic individuals. The upper-left and lower-right triangular regions correspond to scenarios where decision makers discriminate against either Hispanic or White individuals, as indicated by the "$\times$" and "$\cdot$" symbols, respectively; non-discriminatory scenarios are shown by the dashed diagonal line. Red regions indicate where the tests suggest discrimination against Hispanic individuals, blue regions indicate where the tests suggest discrimination against White individuals, and yellow regions indicate where the robust outcome test is inconclusive. Across simulations, the standard outcome test indicates discrimination when, in reality, there is none—and often indicates discrimination against the group that in actuality was favored. In contrast, the robust outcome test is nearly always directionally accurate, though it sometimes returns an inconclusive result.*

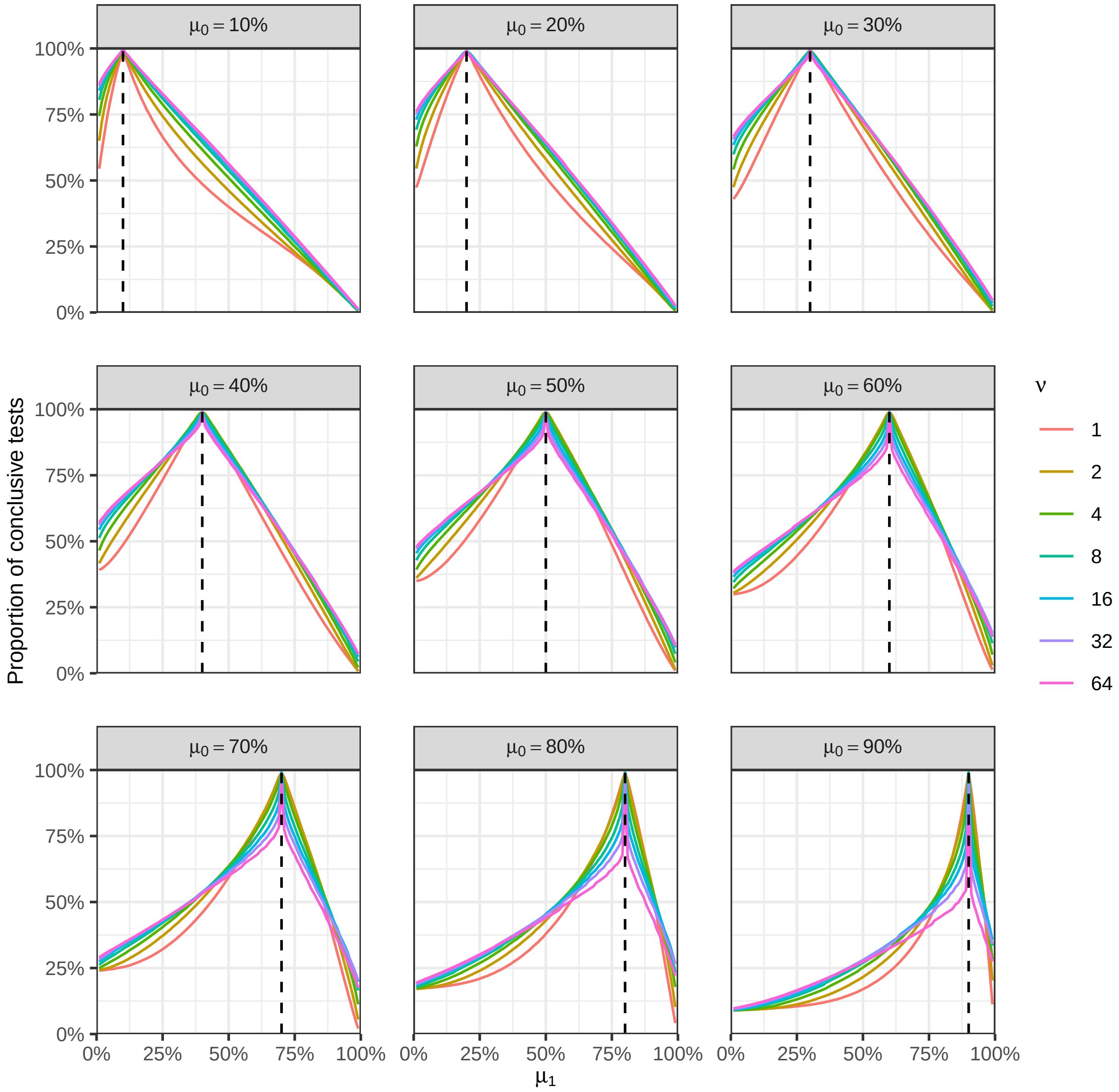

Fig. S7. *Results of a simulation study comparing how often the robust outcome test is conclusive (i.e., indicates that there is discrimination against one of the groups) as a function of the difference between the base rates of the two groups. The facet indicates μ_0, the base rate of group $G = 0$; the x-axis indicates μ_1, the base rate of group $G = 1$; and the y-axis indicates the proportion of scenarios (i.e., choices of t_0 and t_1) in which the robust outcome test is conclusive. Across simulation settings, a larger gap is associated with a greater proportion of inconclusive results, reflecting the reduced sensitivity of the robust outcome test.*

References

1. M Shaked, JG Shanthikumar, *Stochastic Orders.* (Springer), (2007).
2. PR Rosenbaum, DB Rubin, The central role of the propensity score in observational studies for causal effects. *Biometrika* **70**, 41–55 (1983).
3. RD McKelvey, TR Palfrey, Quantal response equilibria for normal form games. *Games Econ. Behav.* **10**, 6–38 (1995).
4. J Keilson, U Sumita, Uniform stochastic ordering and related inequalities. *Can. J. Stat.* **10**, 181–198 (1982).
5. JG Shanthikumar, DD Yao, The preservation of likelihood ratio ordering under convolution. *Stoch. Process. their Appl.* **23**, 259–267 (1986).
6. L Rüschendorf, On conditional stochastic ordering of distributions. *Adv. Appl. Probab.* **23**, 46–63 (1991).
7. W Whitt, Uniform conditional stochastic order. *J. Appl. Probab.* **17**, 112–123 (1980).
8. VI Bogachev, *Measure Theory. Vol. I, II.* (Springer-Verlag, Berlin), pp. Vol. I: xviii+500 pp., Vol. II: xiv+575 (2007).
9. A Guerrero, et al., Ripa board report 2024, (California Department of Justice, Racial Identity and Profiling Act Advisory Board), Technical report (2024).
10. J Angwin, J Larson, S Mattu, L Kirchner, Machine bias in *Ethics of data and analytics.* (Auerbach Publications), pp. 254–264 (2022).
11. Floyd v. City of New York, 959 F. Supp. 2d 540, S.D.N.Y (2013).
12. LF Wightman, LSAC National Longitudinal Bar Passage Study. *LSAC Res. Rep. Ser.* (1998).
13. J Grossman, J Nyarko, S Goel, Reconciling legal and empirical conceptions of disparate impact: An analysis of police stops across California. *J. Law Empir. Analysis* **1** (2024).
14. The People v. William G., 40 Cal.3d 550 (1985).
15. MN Elliott, et al., Using the Census Bureau's surname list to improve estimates of race/ethnicity and associated disparities. *Heal. Serv. Outcomes Res. Methodol.* **9**, 69–83 (2009).
16. T Brennan, W Dieterich, B Ehret, Evaluating the predictive validity of the COMPAS risk and needs assessment system. *Crim. Justice behavior* **36**, 21–40 (2009).
17. S Lansing, New York State COMPAS-probation risk and need assessment study: Examining the recidivism scale's effectiveness and predictive accuracy. *Retrieved March* **1**, 2013 (2012).
18. W Dieterich, T Brennan, WL Oliver, Predictive validity of the COMPAS Core Risk Scales: A probation outcomes study conducted for the Michigan Department of Corrections, (Northpointe Inc., Traverse City, MI), Tech. rep. (2011).
19. D Farabee, S Zhang, RE Roberts, J Yang, COMPAS validation study (2010).
20. AW Flores, K Bechtel, CT Lowenkamp, False positives, false negatives, and false analyses: A rejoinder to "Machine bias: There's software used across the country to predict future criminals. And it's biased against blacks". *Fed. Probat.* **80**, 38 (2016).
21. WA Reich, S Picard-Fritsche, VB Rioja, M Rotter, Evidence-based risk assessment in a mental health court. *Cent. for Court. Innov.* (2016).
22. W Dieterich, C Mendoza, D Hubbard, J Ferro, T Brennan, COMPAS risk scales validation study: An outcomes study conducted for the Santa Barbara County Probation Department, (Northpointe, Traverse City, MI), Tech. rep. (2017).
23. W Dieterich, C Mendoza, D Hubbard, J Ferro, T Brennan, COMPAS risk scales validation study: An outcomes study conducted for the Riverside County Probation Department, (Northpointe, Traverse City, MI), Tech. rep. (2018).
24. Equivant, Practitioner's guide to COMPAS Core (2019) Accessed: 6 March, 2024.
25. Terry v. Ohio, 392 U.S. 1 (1968).
